# Mitochondrial phylogenomics and genetic relationships of closely related pine moth (Lasiocampidae: *Dendrolimus*) species in China, using whole mitochondrial genomes

**DOI:** 10.1186/s12864-015-1566-5

**Published:** 2015-06-04

**Authors:** Jie Qin, Yanzhou Zhang, Xin Zhou, Xiangbo Kong, Shujun Wei, Robert D Ward, Ai-bing Zhang

**Affiliations:** College of Life Sciences, Capital Normal University, Beijing, 100048 China; Institute of Zoology, Chinese Academy of Sciences, 1 Beichen West Road, Chaoyang District, Beijing, 100101 P.R. China; China National GeneBank, BGI-Shenzhen, Beishan Industrial Zone, Yantian District, Shenzhen, Guangdong Province 518083 China; Key Laboratory of Forest Protection, Research Institute of Forest Ecology, Environment and Protection, Chinese Academy of Forestry, State Forestry Administration, No.1 Dongxiaofu, Haidian, Beijing, China; Institute of Plant and Environmental Protection, Beijing Academy of Agriculture and Forestry Sciences, Beijing, 100097 China; CSIRO Oceans and Atmosphere Flagship, GPO Box 1538, Hobart, Tasmania 7001 Australia; College of Life Sciences,Capital Normal University, Beijing, 100048 P. R. China

**Keywords:** Mitochondrial genome, *Dendrolimus punctatus*, *D. tabulaeformis*, *D. spectabilis*, Subspecies

## Abstract

**Background:**

Pine moths (Lepidoptera; Bombycoidea; Lasiocampidae: *Dendrolimus* spp.) are among the most serious insect pests of forests, especially in southern China. Although COI barcodes (a standardized portion of the mitochondrial cytochrome c oxidase subunit I gene) can distinguish some members of this genus, the evolutionary relationships of the three morphospecies *Dendrolimus punctatus, D. tabulaeformis* and *D. spectabilis* have remained largely unresolved. We sequenced whole mitochondrial genomes of eight specimens, including *D. punctatuswenshanensis*. This is an unambiguous subspecies of *D. punctatus*, and was used as a reference for inferring the relationships of the other two morphospecies of the *D. punctatus* complex. We constructed phylogenetic trees from this data, including twelve published mitochondrial genomes of other Bombycoidea species, and examined the relationships of the *Dendrolimus* taxa using these trees and the genomic features of the mitochondrial genome.

**Results:**

The eight fully sequenced mitochondrial genomes from the three morphospecies displayed similar genome structures as other Bombycoidea species in terms of gene content, base composition, level of overall AT-bias and codon usage. However, the *Dendrolimus* genomes possess a unique feature in the large ribosomal 16S RNA subunits (rrnL), which are more than 60 bp longer than other members of the superfamily and have a higher AC proportion. The eight mitochondrial genomes of *Dendrolimus* were highly conservative in many aspects, for example with identical stop codons and overlapping regions. But there were many differences in start codons, intergenic spacers, and numbers of mismatched base pairs of tRNA (transfer RNA genes).

Our results, based on phylogenetic trees, genetic distances, species delimitation and genomic features (such as intergenic spacers) of the mitochondrial genome, indicated that *D. tabulaeformis* is as close to *D. punctatus* as is *D. punctatus wenshanensis*, whereas *D. spectabilis* evolved independently from *D. tabulaeformis* and *D. punctatus*. Whole mitochondrial DNA phylogenies showed that *D. spectabilis* formed a well-supported monophyletic clade, with a clear species boundary separating it from the other congeners examined here. However, *D. tabulaeformis* often clustered with *D. punctatus* and with the subspecies *D. punctatus wenshanensis*. Genetic distance analyses showed that the distance between *D. tabulaeformis* and *D. punctatus* is generally less than the intraspecific distance of *D. punctatus* and its subspecies *D. punctatus wenshanensis*. In the species delimitation analysis of Poisson Tree Processes (PTP), *D. tabulaeformis*, *D. punctatus* and *D. punctatus wenshanensis* clustered into a putative species separated from *D. spectabilis*. In comparison with *D. spectabilis*, *D. tabulaeformis* and *D. punctatus* also exhibit a similar structure in intergenic spacer characterization. These different types of evidence suggest that *D. tabulaeformis* is very close to *D. punctatus* and its subspecies *D. punctatus wenshanensis*, and is likely to be another subspecies of *D. punctatus.*

**Conclusions:**

Whole mitochondrial genomes possess relatively rich genetic information compared with the traditional use of single or multiple genes for phylogenetic purposes. They can be used to better infer phylogenetic relationships and degrees of relatedness of taxonomic groups, at least from the aspect of maternal lineage: caution should be taken due to the maternal-only inheritance of this genome. Our results indicate that *D. spectabilis* is an independent lineage, while *D. tabulaeformis* shows an extremely close relationship to *D. punctatus*.

**Electronic supplementary material:**

The online version of this article (doi:10.1186/s12864-015-1566-5) contains supplementary material, which is available to authorized users.

## Background

Insect mitochondrial genomes are usually a closed-circular molecule 14-20 kilo base pairs (kbp) in size [1]. They contain 13 protein-coding genes (PCGs), 22 transfer RNAs (tRNAs), two ribosomal RNAs (rRNAs), and a large non-coding region called the control region (CR, or the AT-rich region) which includes the essential regulatory elements for transcription and replication [1,2]. Due to their unique characters, which include small genome size, easily accessible nature, faster nucleotide substitution rates and the presence of strictly orthologous genes, mitochondrial genomes have been widely used as molecular markers for phylogenetic analyses and the investigation of questions concerning comparative and evolutionary genomics [3-10]. Whole mitochondrial genomes provide not only more general genetic information than shorter sequences of individual genes such as the COI gene, but also sets of genome-level characters, such as the relative position of different genes, structural genomic features and compositional features [1,2,11,12]. Whole mitochondrial genomes are widely used to infer phylogenetic relationships at different taxonomic levels [7,13-18]. Furthermore, mitochondrial genomes often evolve faster through higher mutation rates than nuclear genomes, especially in intergenic regions [19,20], and can be powerful markers for the inference of phylogenetic relationships among closely related taxa [9,21-24].

*Dendrolimus* species (Lepidoptera, Lasiocampidae) are the most serious phytophagous pests worldwide. *Dendrolimus pini* causes destructive damage of the Scots pine (*Pinus sylvestris*) in Europe [25], and *Dendrolimus* moths cause extensive forest damage in China [26-31]. The occurrence of different species of these pine moths and the existence of natural hybrids leads to heterosis and strong tolerance to pesticides, giving high survival rates and making them difficult to eradicate [27]. The need for improved biological pest control means that it is very important to elucidate the genetic relationships of pine moth species. However, three (*D. punctatus* [32], *D. tabulaeformis* [33], *D. spectabilis* [34]) of the six commonly occurring *Dendrolimus* species in China cannot be readily discriminated, and their taxonomic status is uncertain. Previous studies revealed that these three can hybridize, and it has been suggested that *D. tabulaeformis* and *D. spectabilis* are subspecies of *D. punctatus* [26,27], although other studies treat all three as different species [29-31]. A recent extensive molecular systematic study, looking at mitochondrial COI and several nuclear genes, used different phylogenetic and DNA barcoding methods to assess this complex. The phylogenetic relationships of *D. punctatus, D. tabulaeformis, D. spectabilis* could not be fully resolved, although their close relationship was confirmed [35]. This species group thus provides a good model for investigating the utility of the mitochondrial genome in exploring relationships of closely related species groups.

To clarify the taxonomic and phylogenetic relationships of these species, we report here eight complete mitochondrial genomes of the three morphospecies *Dendrolimus punctatus*, *D. tabulaeformis* and *D. spectabilis*. We compare their genome structures in detail and analyze the relationships between them.

## Methods

### Specimen collection, DNA extraction, PCR amplification, cloning and sequencing

Eight adults of three morphospecies (*D. punctatus* (2), *D. punctatus wenshanensis* (2), *D. tabulaeformis* (2) and *D. spectabilis* (2)) were collected from five locations (Additional file 1). A taxonomic expert (Professor Chun-sheng Wu, Institute of Zoology, Chinese Academy of Sciences) used traditional morphological approaches to identify these specimens. All specimens were preserved in 95% ethanol and maintained at 4°C for long term storage. DNA was isolated from thoracic muscle tissue using the DNeasy Blood and Tissue kit (QIAGEN) following the manufacturer’s protocol.

Mitochondrial genomes were PCR-amplified and sequenced by Sanger sequencing. Whole genomes of the eight specimens were mainly assembled from 14 overlapping PCR fragments (Additional file 2). Eight pairs of universal primers were selected and used for initial amplifications [3,4]. From these sequences, six species-specific primer sets were designed to amplify remaining sections. A specific fragment of DNA was amplified using universal primers by the following conditions: an initial denaturation at 94°C for 4 min, followed by 40 cycles of denaturation at 94°C for 30 s, annealing at 45–55°C for 30 s, elongation at 72°C for 45 s, and a final extension step of 72°C for 10 min. Species-specific primers were amplified with long PCR reaction conditions: an initial denaturation at 92°C for 2 min, followed by 40 cycles of denaturation at 92°C for 30 s, annealing at 45–55°C for 30 s, elongation at 60°C for 12 min, and a final extension step of 60°C for 20 min. All reactions were performed using Takara LA taq (TaKaRa Co., Dalian, China). PCR fragments containing the control region could not be sequenced directly; they were cloned into the pEASY-T3 Cloning Vector (Beijing TransGen Biotech Co., Ltd., Beijing, China) and then sequenced by M13-F (CGCCAGGGTTTTCCCAGTCACGAC) and M13-R (GAGCGGATAACAATTTCACACAGG) primers.

### Sequence assembly and annotation

Raw sequences were checked manually by eye using BioEdit [36] and assembled using the SeqMan program from the laser gene package DNAStar (Madison, USA). The tRNA genes were identified by tRNAscan-SE Search Server v.1.21 [37], with source set for Mito/Chloroplast and Genetic Code for tRNA Isotype Prediction set for Invertebrate Mito. To detect overall tRNA genes, the cove cutoff score could be set lower (≥1). The locations of protein-coding and rRNA genes were determined by comparing homologous sequences with other published Lepidoptera mitochondrial sequences downloaded from GenBank.

### Evolutionary relationships based on phylogenetic analysis

There has been much controversy concerning the evolutionary relationships of closely related species of *Dendrolimus* [26-31]. The three morphospecies *D. spectabilis*, *D. punctatus* and *D. tabulaeformis* can hybridize with each other. Furthermore, *D. punctatus* and *D. tabulaeformis* have highly similar diets and morphological features. Different geographical environments and host plants can result in varied morphology, and *D. spectabilis* and *D. tabulaeformis* have been treated as subspecies of *D. punctatus* [26-28]. We assessed their genetic relationships using whole mitochondrial genome data (including genomic features), with *D. punctatus wenshanensis* (an unambiguous subspecies of *D. punctatus*) as a baseline reference to infer relationships between the two ambiguous morphospecies (*D. tabulaeformis* and *D. spectabilis*) and *D. punctatus*.

Phylogenetic analyses were performed on 20 complete mitochondrial genomes of Bombycoidea. Eight mitochondrial genomes were newly sequenced in this study (Table 1) and 12 others downloaded from GenBank. Two Geometroidea species were used as outgroups: *Biston panterinaria* (NC_020004) [38] and *Phthonandria atrilineata* (NC_010522) [39]. Nucleotide sequences of the 13 protein-coding genes were aligned using a perl script on the basis of protein alignments. The basic algorithm firstly translated DNA sequences into proteins, the protein sequences were then aligned, and finally the DNA sequences were aligned on the protein sequences. This method generated a robust DNA sequence alignment of both distantly and closely related species, as direct DNA sequence alignment may produce unreliable alignments by introducing many indels (gaps). The pipeline can be provided on request (at email zhangab2008@gmail.com or zhangab2008@mail.cnu.edu.cn). The tRNA and rRNA genes were aligned using MUSCLE [40]. Individual genes and aligned partitions were concatenated with SequenceMatrix [41]. Two datasets were generated to infer phylogenetic relationships. One included 13 protein-coding genes (13PCGs), the other 37 genes, including 13 protein coding genes, 22 transfer RNA genes and two ribosomal RNA genes (37gene). In order to improve the reliability of our phylogenetic analyses, several partitions were set in the two datasets. The first dataset was divided into 13 partitions for each protein coding gene, and the second dataset was divided into 15 partitions: each protein-coding gene, the concatenated 22 tRNA genes, and the concatenated rRNA genes. PartitionFinder software was subsequently used to select the optimal scheme and the best-fitting substitution model for each partition set under the Bayesian Information Criterion [42]. Two datasets were analyzed by both Maximum likelihood (ML) and Bayesian Inference (BI) methods [43,44].

**Table 1:** **List of taxa analyzed in this study**

Family	Species	Acc.number	Reference
Bombycidae	*Rondotia menciana*	NC_021962	Kong,W.Q.unpublished
	*Bombyx mori*	NC_002355	Lee et al., unpulished
	*Bombyx mandarina*	NC_003395	[41]
Sphingidae	*Sphinx morio*	NC_020780	[42]
	*Manduca sexta*	NC_010266	[43]
Saturniidae	*Samia cynthia ricini*	NC_017869	[44]
	*Eriogyna pyretorum*	NC_012727	[45]
	*Antheraea pernyi*	NC_004622	[46]
	*Attacus atlas*	NC_021770	Liu,Y.-Q., unpulished
	*Antheraea yamamai*	NC_012739	[47]
	*Saturnia boisduvalii*	NC_010613	[48]
	*Actias selene*	NC_018133	[49]
Lasiocampidae	*Dendrolimus spectabilis02*	KJ_913815	In this study
	*Dendrolimus spectabilis13*	KJ_913816	In this study
	*Dendrolimus punctatus04*	KJ_913813	In this study
	*Dendrolimus punctatus05*	KJ_913814	In this study
	*Dendrolimus tabulaeformis06*	KJ_913817	In this study
	*Dendrolimus tabulaeformis38*	KJ_913818	In this study
	*Dendrolimus punctatus wenshanensis03*	KJ_913811	In this study
	*Dendrolimus punctatus wenshanensis06*	KJ_913812	In this study

The ML analyses were conducted using RaxML [43] with the selected partition scheme (Additional file 3). Because different partition models cannot be set in RaxML, GTRGAMMAI was selected for both the 13PCGs dataset and the 37genes dataset. In RaxML, the rapid hill-climbing algorithm starting from 100 randomized maximum-parsimony trees was used for ML searches. Confidence values of the ML trees were evaluated via bootstrap tests with 1000 iterations.

The BI analyses were conducted using MrBayes ver.3.1 [44] with the best partition scheme (Additional file 3). All BI analyses were conducted with two sets of four Markov chains (one cold and three hot chains). Three criteria were used to verify convergence of BI analyses in order to be confident about the reliability of the results: (1) the average standard deviation of split frequencies was less than 0.01, (2) the potential scale reduction factors (PSRF) [45] were close to 1.0 for all parameters, and (3) the effective sample size value in tracer software exceeded 200 [46]. Each set was sampled with a burnin of 25%. Bayesian posterior probabilities were estimated as the confidence values of the BI tree (BBP).

### Evolutionary relationships based on genome analysis and genetic distance calculations

Genome analysis was performed with the 20 mitochondrial sequences of Bombycoidea (Table 1). Nucleotide compositions, codon usage (excluding stop codons) and Relative Synonymous Codon Usage (RSCU) were calculated with MEGA 4.0 [47]. Composition skew analysis used the formulae AT skew = [A-T]/[A + T] and GC skew = [G-C]/[G + C] [48].

**Figure 1:**
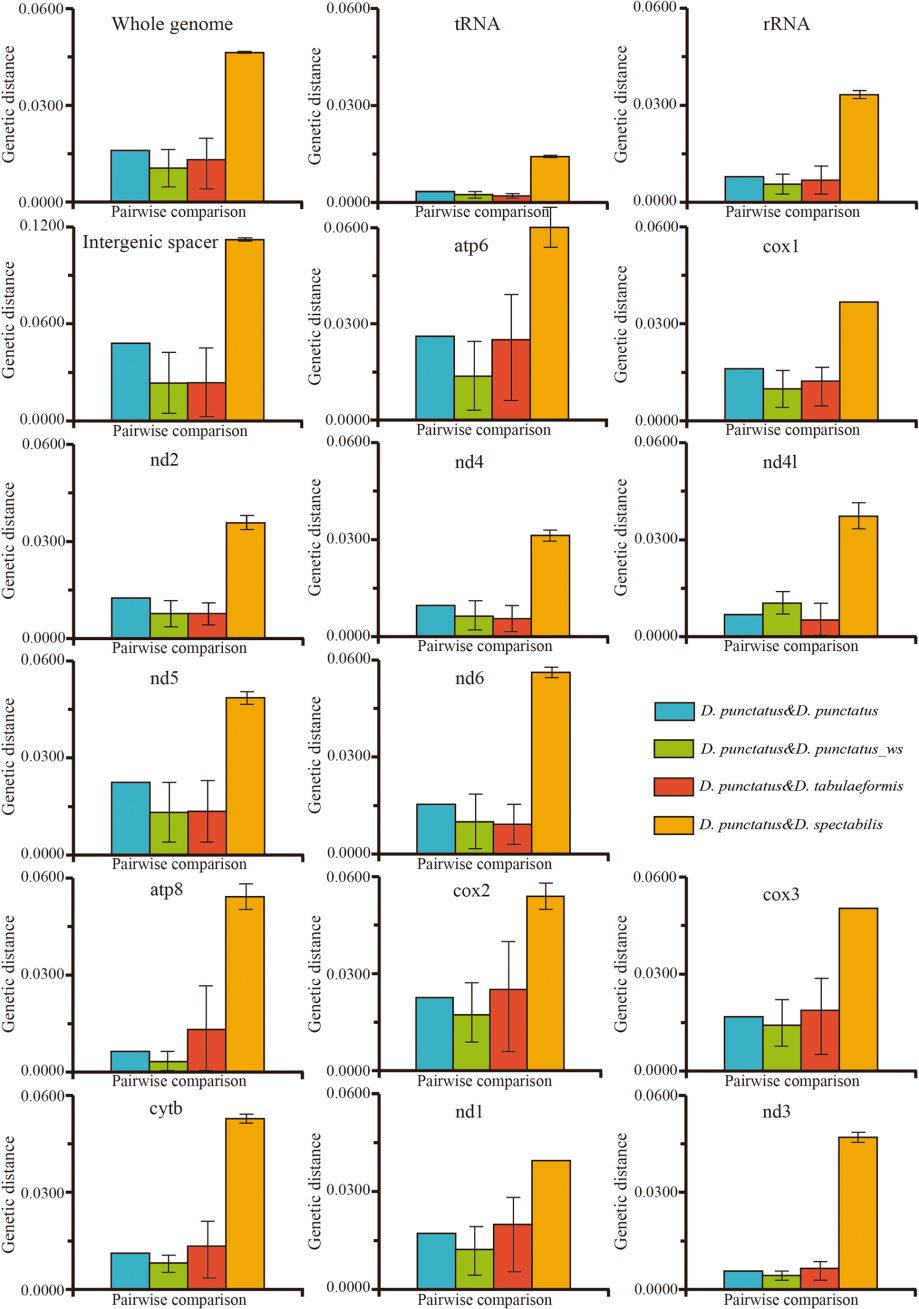
Average K2P genetic distances for different genes and regions of: intraspecific *D. punctatus* (blue histogram), and genetic distances of *D. tabulaeformis* to *D. punctatus* (red), *D. punctatus wenshanensis* to *D. punctatus* (green), and *D. spectabilis* to *D. punctatus* (yellow) for different genes and regions. Bars show range of values.

Genetic distance is generally considered to be an important aspect of species discrimination. A clear gap in genetic distance between intraspecific and interspecific variation usually indicates a species boundary [49,50,35]. To explore the relationships of *D. punctatus, D. tabulaeformis* and *D. spectabilis,* we calculated Kimura's two parameter (K2P) genetic distances among different genes and regions using MEGA 5.0[51] (Figure 1). We calculated pairwise distances among all eight individuals of *D. punctatus*, *D. spectabilis*, *D. tabulaeformis* and *D. punctatus wenshanensis*, for different genes and regions (Additional file 4). Then average genetic distances (with range) were calculated. Genetic distances of *D. punctatus* to *D. tabulaeformis*, and *D. punctatus* to *D. spectabilis,* were then compared with the intraspecific distance of *D. punctatus* and with the genetic distance of *D. punctatus* and its recognized subspecies *D. punctatus wenshanensis*.

A second investigation used the COI gene alone, but from much larger sample sizes ((*D. punctatus* (195)*, D. tabulaeformis* (6)*, D. spectabilis* (14)*, D. punctatus wenshanensis* (35)) and different geographical populations. K2P genetic distances were calculated with a Perl script [11]. Confidence intervals were calculated using R with t.test function in a stats package ( http://cran.r-project.org/).

### Evolutionary relationships based on species delimitation

We applied the Poisson Tree Processes (PTP) model to delimit species on a rooted phylogenetic tree for our two datasets (13PCGs and 37gene) [52]. The PTP program models speciation or coalescent events relative to number of substitutions represented by branch lengths, and uses heuristic algorithms to estimate the most likely classification of branches into population and species-level processes. It does not require ultrametrization of trees and outperforms the commonly used General Mixed Yule Coalescent (GMYC) model when species are closely related and evolutionary distances small [52,53]. The BI phylogenetic trees obtained with MrBayes 3.1 [44], described previously, were used as input trees. PTP analyses were run from Python using the ETE (Python Environment for Tree Exploration) package [54] for tree manipulation and visualization.

## Results

### Mitochondrial genome organization

Eight complete mitochondrial genomes from *Dendrolimus* were sequenced, annotated and deposited in GenBank (Table 1). Genome organizations are presented in an additional file (Additional file 5 a-h). All genomes contained the same set of 37 genes (13 protein-coding genes, 22 tRNA genes, and two rRNA genes) and a putative control region [1]. Genome sizes ranged from 15,407 bp to 15,419 bp, and all exhibited similar sequence characteristics. The gene order of these eight pine moths was identical to other ditrysian lepidopterans with the trnM gene location type (trnM-trnI-trnQ) [55-57]. All 13 PCGs start with typical ATN codons, except for cox1 which used CGA. Nine genes (nad2, atp8, atp6, cox3, nad5, nad4l, nad6, cob, nad1) share the same complete termination codon TAA, and four genes use incomplete stop codons (a single T for cox1, cox2 and nad4, TA for nad3) (Additional file 6). Incomplete stop codons are common in lepidopteran mitochondrial genomes and are presumed to be completed via post-transcriptional polyadenylation [58,59].

As for other Lepidoptera, 22 tRNA genes were detected in *Dendrolimus* mitochondrial genomes, and ranged in size from 64 to 71 bp. Fourteen tRNA genes were coded on the majority strand (J-strand) with eight coded on the minority strand (N-strand). All the tRNA genes have classic cloverleaf secondary structures except for the trnS1 (AGN) gene, where the dihydrouridine (DHU) arm is replaced by unpaired nucleotides. This feature is common to most Lepidopteran mitochondrial genomes except the tortricid *Adoxophyes honmai*, which has all tRNA genes with complete cloverleaf structures [60]. These aberrant tRNA genes may be modified via RNA-editing mechanisms [61,62]. Many mismatches were found in each of the newly sequenced genomes resulting in some small differences (Additional file 7). There were 16 identical mismatched base pairs and G-U wobble pairs in tRNA structures for all eight samples.

Base composition, AT-skew and codon usage were calculated for the eight newly sequenced genomes and the 12 published Bombycidae mitochondrial genomes (Table 1). The base composition of the J-strand of the *Dendrolinus* species fell within the range of other Bombycoidea species, and their nucleotide compositions were significantly biased toward A and T (Additional file 8). The nucleotide skew statistics for the entire majority strand of eight individuals indicated a weak A skew and a moderate C skew. In the protein coding genes, both the second and the third positions had negative AT-skew and GC-skew, with the first position having positive AT-skew and GC-skew; overall, PCGs had a negative AT-skew and a positive GC-skew (Additional file 8). The nucleotide skew statistics for the tRNA genes of the eight *Dendrolimus* individuals indicated a weak A skew and a moderate C skew. The rrnL gene of *Dendrolimus* is more than 60 bp longer than that of other Bombycoidea species, and the nucleotide skew statistics for rrnL of the eight individuals showed higher A- and C-skews than other Bombycoidea.

Codon usage and RSCU results of our eight moths and the other Bombycoidea species are also given (Additional file 9). Codon usage bias has been detected in currently sequenced mitochondrial genomes. Excluding stop codons, there were 3726 codons in both *D. spectabilis* and *D. tabulaeformis*,. One additional codon was detected in *D. punctatus*. The codon families show very similar behavior among all 20 species considered (Additional file 9). In the eight newly sequenced genomes, eight codon families (Phe, Leu2, Ile, Met, Ser2, Tyr, Asn, Gly) had at least 50 codons per thousand codons (CDs), and two codon families (Leu2, Ile) had at least 100 codons per thousand CDs; other Bombycoidea species had three codon families (Phe, Leu2, Ile) with at least 100 codons per thousand CDs.

RSCU results of Bombycoidea showed that codons exhibit a strong AT-bias in the third codon position, and GC-rich codons are less preferred among the usage of both four- and twofold degenerate codons [63] (Additional file 9). In our eight specimens, many codons were absent, with AGG absent in *D. punctatus wenshanensis03, D. punctatus wenshanensis 06, D. punctatus04, D. tabulaeformis06* and *D. tabulaeformis38*; UGC and AGG were absent in *D. spectabilis02* and *D. spectabilis13* (Additional file 9).

### Evolutionary relationships based on phylogenetic analyses

**Figure 2:**
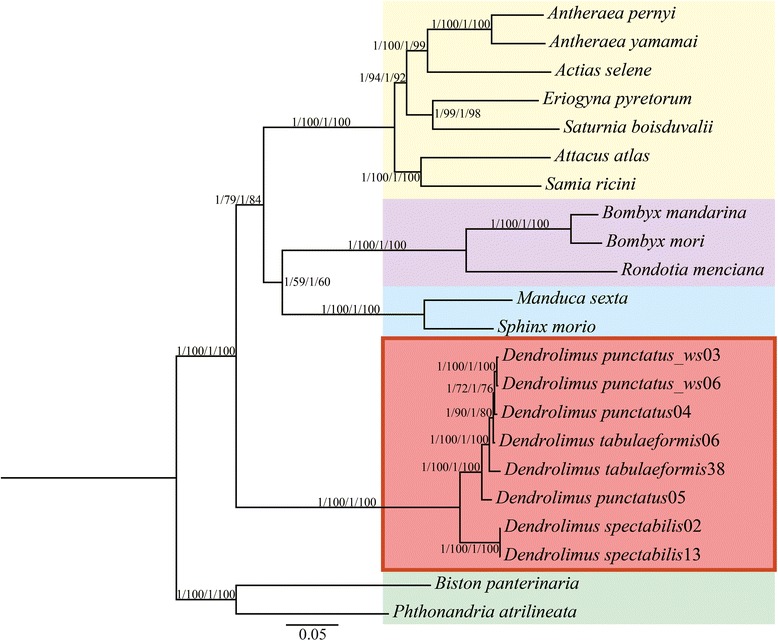
Phylogenetic tree (ML and BI) of Bombycoidea species with outgroups constructed with (1) 13 protein coding genes (13PCGs); (2) 37 genes (13 protein-coding genes + 22 transfer RNA genes + 2 ribosomal RNA genes, 37gene). Numbers above or below branches indicate bootstrap values, in the order of presentation 13PCGs-BI/13PCGs-ML/37gene-BI/37gene-ML. Clades with different colors indicate different families. Specimens within the red box are the newly sequenced genomes of this study.

The phylogenetic analyses conducted in this study yielded the same topological relationships for the 13PCGs and 37gene data matrices in both ML and BI trees (Figure 2). The BI analyses met the three criteria simultaneously to ensure accurate results. Phylogenetic trees showed that in the genus *Dendrolimus*, the morphospecies *D. spectabilis* formed a stable clade distinct from other *Dendrolimus*, and that *D. punctatus* (including *D. punctatus wenshanensis*) and *D. tabulaeformis* formed a separate monophyletic clade. Thus from a phylogenetic point of view, we conclude that *D. spectabilis* has evolved as an independent lineage distinct from that of *D. punctatus* and *D. tabulaeformis.*

### Evolutionary relationships based on genetic distance: individual genes and regions versus whole mitochondrial genome analysis

In most genes and regions (such as intergenic spacers, tRNA genes, rRNA genes, whole genome, atp6, cox1, nd2, nd4, nd4l, nd5 and nd6), the average K2P distances of *D. punctatus* to *D. tabulaeformis* were less than the K2P intraspecies distances of *D. punctatus* to *D. punctatus* or the distances of *D. punctatus* to its subspecies *D. punctatus wenshanensis* (Additional file 4). But for the atp8, cox2, cox3, cytb, nd1 and nd3 genes, the average genetic distances of *D. punctatus* to *D. tabulaeformis* were higher than the average intraspecific distances of *D. punctatus* and distances of *D. punctatus* to *D. punctatus wenshanensis* (Figure 1). However, for most regions and genes, especially for the complete genome, genetic distances between *D. punctatus* and *D. tabulaeformis* were less than the intraspecific differences of *D. punctatus*. These findings suggest that *D. punctatus* and *D. tabulaeformis* are extremely similar. The average K2P interspecific distances of *D. punctatus* and *D. spectabilis,* based on whole genomes or genes or regions, were higher than the K2P intraspecific distances of *D. punctatus* and distances of *D. punctatus* to *D. punctatus wenshanensis* (Figure 1). These results therefore suggest that *D. spectabilis* is a relatively distinct lineage from *D. punctatus* and *D. tabulaeformis*.

**Figure 3:**
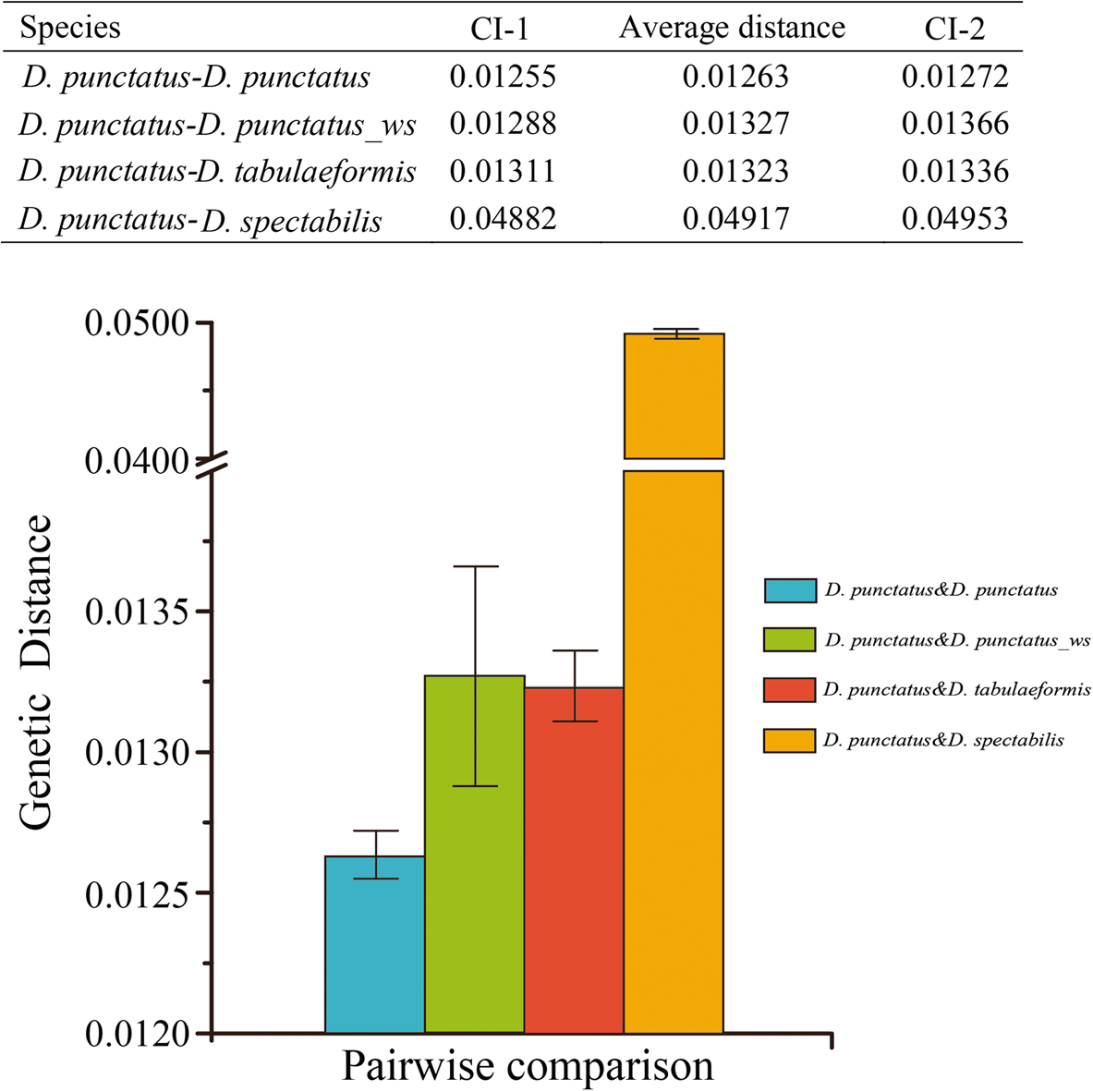
Average intraspecific K2P genetic distances of *D. punctatus* and genetic distances of *D. tabulaeformis* to *D. punctatus*, *D. punctatus wenshanensis* to *D. punctatus* and *D. spectabilis* to *D. punctatus* for the COI barcoding region of cox1. CI-1 and CI-2 indicate 95% confidence intervals. The length of line in the bar graph shows the confidence intervals. Note that large numbers of specimens were used for this assessment.

Another dataset, comprising many more specimens of *D. punctatus*, *D. spectabilis* and *D. tabulaeformis*, but only assessed for the barcoding gene region COI, was also examined (Figure 3). The average K2P genetic distance of *D. spectabilis* and *D. punctatus* (0.0491) was significantly higher than the intraspecific distance of *D. punctatus* (0.0126) and the genetic distances of *D. punctatus* to *D. tabulaeformis* (0.0132) and to *D. punctatus wenshanensis* (0.0133). The COI genetic distance between *D. punctatus* and *D. tabulaeformis* is similar to the distance between *D. punctatus* and its subspecies *D. punctatus wenshanensis,* demonstrating that *D. tabulaeformis* has, if anything, a closer relationship to *D. punctatus* than does *D. punctatus wenshanensis.*

Note that these analyses were also repeated with the best fitting nucleotide substitution models (see Additional files 10 and 11), and these yielded the same conclusions as the K2P model [64].

However, there were some differences between the two datasets, the one using just eight samples but sequenced for the entire cox1 gene and the other examining many more samples but only for the COI region of cox1. In the mitochondrial genome dataset, the results from the cox1 gene show that the average genetic distance of *D. punctatus* to *D. tabulaeformis* was higher than the average genetic distance of *D. punctatus* to *D. punctatus wenshanensis* but less than the intraspecific distance of *D. punctatus*. In the larger COI dataset, the average interspecific distance of *D. punctatus* and *D. tabulaeformis* was higher than average intraspecific distance of *D. punctatus* but less than the genetic distance of *D. punctatus* to *D. punctatus wenshanensis*. There are two possible causes of this discrepancy: firstly, sample sizes are small for the cox1 dataset so that confidence intervals cannot be estimated and compared, and secondly, the cox1 gene in the mitochondrial genome dataset was 1,531 bp long, much longer than the length of the COI barcode sequence examined here (588 bp).

### Evolutionary relationships based on species delimitation

**Figure 4:**
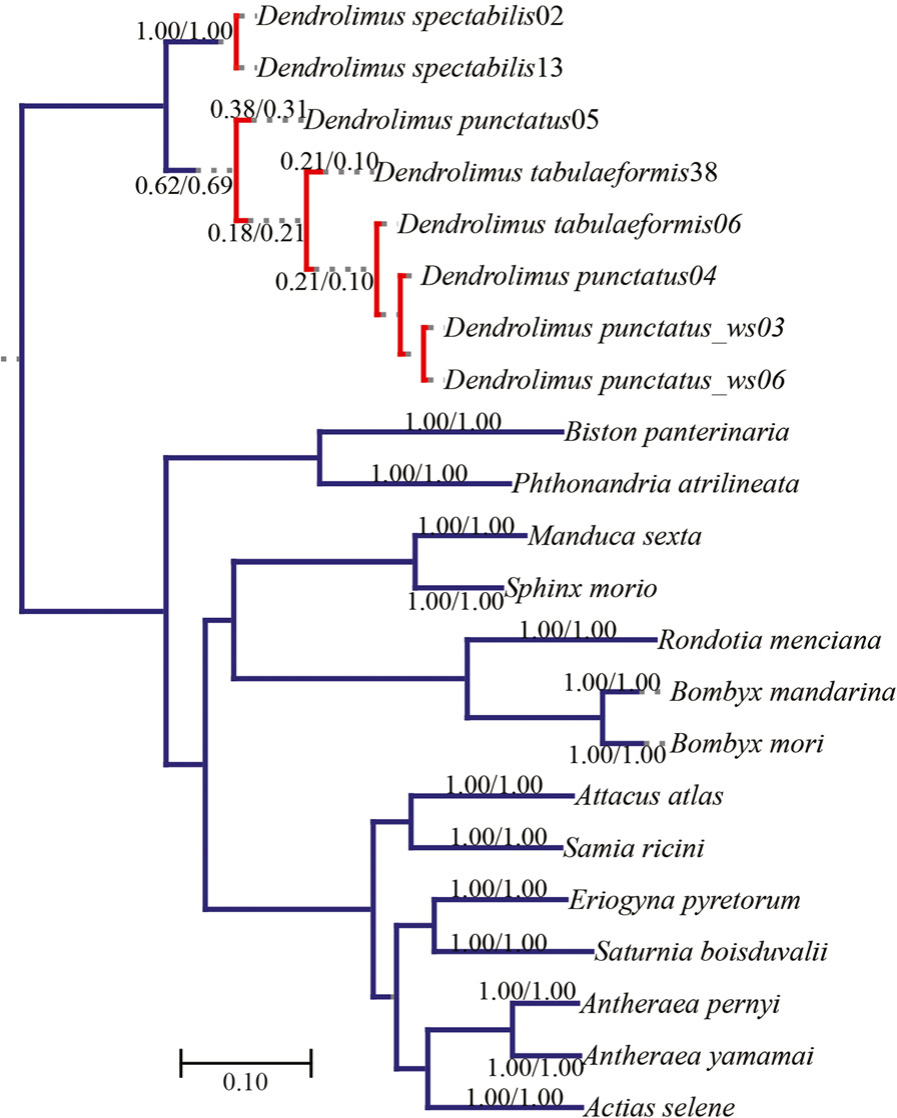
Species-delimitation using the PTP model conducted with (1) 13 protein coding genes (13PCGs) and (2) 37 genes (13 protein-coding genes + 22 transfer RNA genes + 2 ribosomal RNA genes, 37gene). Numbers above or below branches indicate support, in the order of presentation 13PCGs/37gene.

The PTP model identified a total of 14 putative species from the 20 Bombycoidea genomes (Figure 4). Except for the eight newly sequenced genomes of *Dendrolimus*, they all formed independent entities. Two individuals of *D. spectabilis* clustered together forming a monophyletic group, while the other *Dendrolimus* species clustered in a separate clade as a single species (Figure 4). Coincident with the results of phylogenetic analyses, *D. punctatus* and *D. tabulaeformis* can be considered as one putative species and *D. spectabilis* as another.

### Evolutionary relationships with a mitochondrial genome feature: intergenic spacers

Mitochondrial genomes possess many organizational characteristics. The intergenic regions, for example, do not encode genes and as a result are under more relaxed selection and evolve faster than coding genes [19,20]. Consequently, intergenic regions are expected to be more variable than coding genes, and therefore could be a useful feature when studying the relationships of closely related species.

There were seventeen intergenic spacers in the eight sequenced mitochondrial genomes (Additional file 12). Six were longer than 10 bp: trnM-nd2, trnY-cox1, atp6-cox3, trnA-trnR, trnN-trnS (AGN) and nd4-nd4l. Comparing these six regions of the eight individuals, many differences can be observed (Additional file 13). The intergenic spacer trnM-nd2 was the largest at 58 bp, and is common to most lepidopteran mitochondrial genomes. In this spacer, the two individuals of *D. spectabilis* had identical nucleotide patterns. Except for one specimen, *D. punctatus*, D*. punctatus wenshanensis* and *D. tabulaeformis* were also identical, but differed by two nucleotides from *D. spectabilis*. The exception was *D. punctatus04,* with two different bases. In the other five intergenic spacers, the two *D. spectabilis* always showed an identical nucleotide distribution, which differed from those of *D. punctatus*, *D. punctatus wenshanensis* and *D. tabulaeformis*. The latter taxa present a mainly similar nucleotide pattern, but *D. punctatus04* and *D. punctatus05* often showed some differences. In the trnY-cox1 region, *D. punctatus05* has 11 nucleotide differences, and for the atp6-cox3, trnA-trnR, trnN-trnS(AGN) and nd4-nd4l regions, *D. punctatus04* showed one to three nucleotides difference. *D. tabulaeformis* always showed an identical nucleotide pattern to *D. punctatus wenshanensis.* These findings further support the conclusions that *D. tabulaeformis* has as close a relationship with *D. punctatus* as has *D. punctatus wenshanensis,* and that *D. spectabilis* is distinct.

## Conclusions and Discussion

In this study, whole mitochondrial genomes were sequenced using conventional Sanger sequencing with the Primer-Walking method being applied to long fragment sequencing. Sequences were corrected manually to improve the accuracy of sequence determination, and assembled using overlap regions to obtain full-length mitochondrial genomes. Even though earlier laboratory hybridization studies suggested that both the current morphospecies *D. spectabilis* and *D. tabulaeformis* are subspecies of *D. punctatus* [26,27], there is as yet no consensus on the exact relationships of the three taxa [29-31]. After assessing the whole mitochondrial genome data in multiple ways, we find that *D. spectabilis* forms an independent lineage from *D. punctatus*, while *D. tabulaeformis* has a very close relationship with *D. punctatus*. Like *D. punctatus wenshanensis*, *D. tabulaeformis* is likely to be a subspecies of *D. punctatus*, and this conclusion is also supported by an earlier DNA barcoding based study [35].

Geographically, *D. spectabilis* is distributed in northern China, and its range does not overlap with *D. punctatus* (central and southern China). Both genetic and distributional data strongly suggest that *D. spectabilis* has evolved as a separate lineage or species, perhaps recently. In contrast, *D. tabulaeformis* is mostly distributed in central China and geographically has not totally separated from *D. punctatus*. The eight specimens that formed the basis of this study were selected from different locations (Additional file 1): two individuals of *D. punctatus* from Baise, Guangxi, two *D. punctatus wenshanensis* from Wenshan Shilin, Yunnan, two *D. tabulaeformis* from Chengde, Hebei, *D. spectabilis*02 from Tongliaokulun, Neimenggu and *D. spectabilis*13 from Taian, Shandong. *D. punctatus, D. punctatus wenshanensis* and *D. tabulaeformis* came from different sampling sites, but still have very high sequence similarity, and genetically cannot be unambiguously distinguished. However, the two individuals of *D. spectabilis,* although from different sampling sites, showed a high degree of sequence identity to one another and significant differences from *D. punctatus, D. punctatus wenshanensis* and *D. tabulaeformis.*

The main goal of this study was to explore the phylogenetic relationships of closely related species in the genus *Dendrolimus*. The mitochondrial DNA genome was selected because of its relatively faster rate of evolution compared with nuclear markers. However, we note that our study does have several limitations. Firstly, the sample size of each species is relatively small compared with studies where a few molecular markers but many specimens are used. We only studied the whole mitochondrial genome from two specimens of each taxon due to high sequencing costs. An increased sample size would be desirable in future studies. However, we did analyse many more individuals using the COI barcoding region alone, coming to similar conclusions. Secondly, the mitochondrial genome only represents the evolution of maternal lineages, and misleading conclusions may be drawn when there are inconsistencies between mitochondrial and nuclear genealogy. Therefore, the introduction of many, not just one or two, nuclear markers in future studies is also highly desirable. Furthermore, with the development of next-generation sequencing technology, more and more mitochondrial genomes and transcriptomes are being examined to infer phylogenetic relationships [65-67]. We plan to include nuclear markers, including transcriptome analysis, in a future study of this taxon group.

## Additional files

Additional file 1:
**Sample localities of** ***Dendrolinus*** **in China, geographical coordinates and altitudes of collecting sites.**
Additional file 2:
**Mitochondrial DNA regions and their primers used in the present study.**
Additional file 3:
**Partition scheme used in the present study.**
Additional file 4:
**Kimura 2-parameter distance measures for whole mitochondrial genomes and different components.**
Additional file 5:
**(a). Annotation of the mitochondrial genome of** ***D. spectabilis*** **02; (b). Annotation of the mitochondrial genome of** ***D. spectabilis*** **13; (c). Annotation of the mitochondrial genome of** ***D. punctatus*** **04; (d). Annotation of the mitochondrial genome of** ***D. punctatus*** **05; (e). Annotation of the mitochondrial genome of** ***D. punctatus wenshanensis*** **03; (f). Annotation of the mitochondrial genome of** ***D. punctatus wenshanensis*** **06; (g). Annotation of the mitochondrial genome of** ***D.tabulaeformis*** **06; (h). Annotation of the mitochondrial genome of** ***D.tabulaeformis*** **38.**
Additional file 6:
**Start codons and stop codons of the eight sequenced individuals.**
Additional file 7:
**Numbers of mismatched base pairs in the eight sequenced genomes.**
Additional file 8:
**Gene content of our eight sequenced specimens and other published Bombycoidea mitochondrial genomes.**
Additional file 9:
**(a). Codon usage of eight** ***Dendrolimus*** **mitochondrial genomes.** Numbers above the column refer to the number of codons. The total number of codons of each individual is given. CDspT stands for codons per thousand codons. Codon Families are provided on the x axis. (b). The relative synonymous codon usage (RSCU) of eight *Dendrolimus* mitochondrial genomes. Codon Families are provided on the x axis. Codons that are absent in the mitochondrial genomes are marked at the top of columns. (c). Codon usage of Bombycoidea mitochondrial genomes that are published in GenBank. Numbers above the column refer to the number of codons. The total number of codons of each individual is given. CDspT stands for codons per thousand codons. Codon Families are provided on the x axis. (d). Codon usage of Bombycoidea mitochondrial genomes that are published in GenBank. Codon Families are provided on the x axis. Codons that are absent in the mitochondrial genomes are marked at the top of columns. Additional file 10:
**Genetic distance used HKY85 model for whole mitochondrial genomes and different components.**
Additional file 11:
**Genetic distance used selected best-fitting GTR model measures for the COI barcoding region of the additional dataset.**
Additional file 12:
**Intergenic spacer locations and nucleotides of eight sequenced pine moths.**
Additional file 13:
**Six intergenic spacers in the eight sequenced mitochondrial genomes.** The locations of the intergenic spacers are indicated on the top of the figure. The left side of figure is the abbreviated species name. *Spectabilis*02 stands for *Dendrolimus spectabilis*02*, Spectabilis*13 stands for *Dendrolimus spectabilis*13*, Punctatus*04 stands for *Dendrolimus punctatus*04*, Punctatus*05 *stands for Dendrolimus punctatus*05*, Punctatus_ws*03 *stands for Dendrolimus punctatus wenshanensis*03*, Punctatus_ws*06 *stands for Dendrolimus punctatus wenshanensis*06*, Tabulaeformis*06 *stands for Dendrolimus tabulaeformis*06*, Tabulaeformis*38 *stands for Dendrolimus tabulaeformis*38*.* Different colors represent different deoxynucleotides. Green represents adenine, purple guanine, blue cytosine and orange thymine. Dashes (-) indicate no nucleotide.
