# Supplementary material for: Mitochondrial phylogenomics and genetic relationships of closely related pine moth (Lasiocampidae: Dendrolimus) species in China, using whole mitochondrial genomes
Source: BMC Genomics. 2015 Jun 4;16(1):428. doi: 10.1186/s12864-015-1566-5 (PMC4455531; doi:10.1186/s12864-015-1566-5)
Supplement: Additional file 1: — Sample localities of Dendrolinus in China, geographical coordinates and altitudes of collecting sites. [file 12864_2015_1566_MOESM1_ESM.docx]

| Additional file 1 Sample localities of *Dendrolinus* in China, geographical coordinates and altitudes of collecting sites. | | | | |
| --- | --- | --- | --- | --- |
| Specimen (code) | Sample locality | Coordinates | | Altitude (meter) |
|  |  | °N | °E |  |
| *D. tabulaeformis06* (YS06) | Chengde, Hebei | 40.59 ° | 118.53 ° | 816 |
| *D. tabulaeformis38* (YS38) | Chengde, Hebei | 40.59 ° | 118.53 ° | 816 |
| *D. punctatus04* (MW04) | Baise, Guangxi | 23.53° | 106.37° | 243 |
| *D. punctatus05* (MW05) | Baise, Guangxi | 23.53° | 106.37° | 243 |
| *D. punctatus_ws03* (WS03) | Shilin, Yunnan | 24.45° | 103.16° | 1888 |
| *D. punctatus_ws06* (WS06) | Shilin, Yunnan | 24.45° | 103.16° | 1888 |
| *D. spectabilis02* (CS02) | Tongliaokulun, Neimenggu | 42.43 ° | 117.32° | 263 |
| *D. spectabilis13* (CS13) | Taian, Shandong | 36.04° | 117.17° | 488 |
