# Supplementary material for: Mitochondrial phylogenomics and genetic relationships of closely related pine moth (Lasiocampidae: Dendrolimus) species in China, using whole mitochondrial genomes
Source: BMC Genomics. 2015 Jun 4;16(1):428. doi: 10.1186/s12864-015-1566-5 (PMC4455531; doi:10.1186/s12864-015-1566-5)
Supplement: Additional file 2: — Mitochondrial DNA regions and their primers used in the present study. [file 12864_2015_1566_MOESM2_ESM.docx]

| Additional file 2 Mitochondrial DNA regions and their primers used in the present study | | | |
| --- | --- | --- | --- |
| Fragment | Region | Primer(F/R) | Primer sequence(F/R) 5'-3' |
|  |  |  |  |
| F1 | trnW-cox1 | TW-J1301/C1-N2353 | GGTAAAATAAACTAATAATCTTCAAA /GCTCGTGTATCAATATCTATACC |
| F2 | cox1 | C1-J1709/C1-N2776 | AATTGGAGGATTTGGAAATTG /GATAATCTGAATATCGTCGAGG |
| F3 | cox1-cox2 | C1-J2756/C2-N3665 | ACATTTTTTCCACAACATTT /CCACAAATTTCTGAACATTG |
| F4 | cox2-trnK | C2-J3399/TK-N3796 | TCTATCGGACATCAATGATACTG /ACTATAAAATGGTTTAAGAG |
| F5 | cox2-atp6 | Cox2-J3555/Atp6-N4463 | GAACAGTCCCAGCATTAG /TGAATAACCGCAACTGCT |
| F6 | atp6-nad3 | Atp6-J4435/Nad3-N5820 | TTGTGGAGCAAATCATAG /TAGTGGGATAATAGGAAA |
| F7 | nad3-trnN | N3-J5747/TN-N6160 | CCATTTGAATGTGGGTTTGATCC /TCAATTTTATCATTAACAGTGA |
| F8 | nad3-nad5 | Nad3-J5880/Nad5-N7650 | TTCCTATTATCCCACTAT /TTTTATTCTGGGGTTTCT |
| F9 | nad5-nad4 | N5-J7572/N4-N8727 | AAAAGGAATTTGAGCACTTTTAGT /AAATCTTTAATTGCTTATTCTTC |
| F10 | nad4-cytb | Nad4-J8500/Cytb-N11201 | CACGAGAAACCCCAGAAT /CTAATGCGATAACTCCTC |
| F11 | cytb-nad1 | CB-J11335/N1-N12242 | CATATTCAACCCGAATGATA /GTAGCTCAGACTATTTCTTATGA |
| F12 | nad1-rrnS | Nad1-J12110/SR-N14300 | CATAACGATAACGAGGTA /GGCGGTATTTTAGTTCAT |
| F13 | rrnS | SR-J14197/SR-N14745 | TACCTCTACTTTGTTACGACTT /GTGCCAGCAGTTGCGGTTATAC |
| F14 | rrnS-trnY | SR-J14620/trnY-N1435 | TGAAAGTGACGGGCAATA /GACAGTTCACCCAGTTCC |
