# Supplementary material for: Mitochondrial phylogenomics and genetic relationships of closely related pine moth (Lasiocampidae: Dendrolimus) species in China, using whole mitochondrial genomes
Source: BMC Genomics. 2015 Jun 4;16(1):428. doi: 10.1186/s12864-015-1566-5 (PMC4455531; doi:10.1186/s12864-015-1566-5)
Supplement: Additional file 3: — Partition scheme used in the present study. [file 12864_2015_1566_MOESM3_ESM.docx]

| Additional file 3 Partition scheme used in the present study | | |
| --- | --- | --- |
| Subset for 13PCGs | Best model | Subset Partition |
| 1 | GTR+I+G | Atp6/atp8/nd2/nd3/nd6 |
| 2 | GTR+I+G | cox1/cox2/cox3/cytb |
| 3 | GTR+I+G | nd1/nd4/nd4L/nd5 |
| Subset for 37gene | Best model | Subset Partition |
| 1 | GTR+I+G | atp6/atp8/nd2/nd3/nd6 |
| 2 | GTR+I+G | cox1/cox2/cox3/cytb |
| 3 | GTR+I+G | nd1/nd4/nd4L/nd5 |
| 4 | GTR+G | rRNA |
| 5 | GTR+I+G | tRNA |
