# Supplementary material for: Mitochondrial phylogenomics and genetic relationships of closely related pine moth (Lasiocampidae: Dendrolimus) species in China, using whole mitochondrial genomes
Source: BMC Genomics. 2015 Jun 4;16(1):428. doi: 10.1186/s12864-015-1566-5 (PMC4455531; doi:10.1186/s12864-015-1566-5)
Supplement: Additional file 4: — Kimura 2-parameter distance measures for whole mitochondrial genomes and different components. [file 12864_2015_1566_MOESM4_ESM.docx]

| Additional file 4 Kimura 2-parameter distance measures for whole mitochondrial genomes and different components. | | | |
| --- | --- | --- | --- |
| **All** | Range-1 | Average distance | Range-2 |
| *D. punctatus-D. punctatus* | 0.0160 | 0.0160 | 0.0160 |
| *D. punctatus-D. punctatus_ws* | 0.0046 | 0.0105 | 0.0163 |
| *D. punctatus-D. tabulaeformis* | 0.0040 | 0.0131 | 0.0198 |
| *D. punctatus-D. spectabilis* | 0.0460 | 0.0463 | 0.0467 |
| **ATP6** | Range-1 | Average distance | Range-2 |
| *D. punctatus-D. punctatus* | 0.0261 | 0.0261 | 0.0261 |
| *D. punctatus-D. punctatus_ws* | 0.0030 | 0.0137 | 0.0245 |
| *D. punctatus-D. tabulaeformis* | 0.0059 | 0.0250 | 0.0391 |
| *D. punctatus-D. spectabilis* | 0.0538 | 0.0601 | 0.0664 |
| **COX1** | Range-1 | Average distance | Range-2 |
| *D. punctatus-D. punctatus* | 0.0161 | 0.0161 | 0.0161 |
| *D. punctatus-D. punctatus_ws* | 0.0042 | 0.0099 | 0.0156 |
| *D. punctatus-D. tabulaeformis* | 0.0046 | 0.0123 | 0.0166 |
| *D. punctatus-D. spectabilis* | 0.0367 | 0.0367 | 0.0367 |
| **ND2** | Range-1 | Average distance | Range-2 |
| *D. punctatus-D. punctatus* | 0.0125 | 0.0125 | 0.0125 |
| *D. punctatus-D. punctatus_ws* | 0.0036 | 0.0077 | 0.0117 |
| *D. punctatus-D. tabulaeformis* | 0.0043 | 0.0077 | 0.0110 |
| *D. punctatus-D. spectabilis* | 0.0336 | 0.0358 | 0.0381 |
| **ND4** | Range-1 | Average distance | Range-2 |
| *D. punctatus-D. punctatus* | 0.0096 | 0.0096 | 0.0096 |
| *D. punctatus-D. punctatus_ws* | 0.0020 | 0.0063 | 0.0111 |
| *D. punctatus-D. tabulaeformis* | 0.0015 | 0.0055 | 0.0096 |
| *D. punctatus-D. spectabilis* | 0.0295 | 0.0313 | 0.0330 |
| **ND4L** | Range-1 | Average distance | Range-2 |
| *D. punctatus-D. punctatus* | 0.0069 | 0.0069 | 0.0069 |
| *D. punctatus-D. punctatus_ws* | 0.0069 | 0.0104 | 0.0140 |
| *D. punctatus-D. tabulaeformis* | 0.0000 | 0.0052 | 0.0104 |
| *D. punctatus-D. spectabilis* | 0.0333 | 0.0373 | 0.0414 |
| **ND5** | Range-1 | Average distance | Range-2 |
| *D. punctatus-D. punctatus* | 0.0224 | 0.0224 | 0.0224 |
| *D. punctatus-D. punctatus_ws* | 0.0040 | 0.0132 | 0.0224 |
| *D. punctatus-D. tabulaeformis* | 0.0040 | 0.0135 | 0.0230 |
| *D. punctatus-D. spectabilis* | 0.0466 | 0.0486 | 0.0505 |
| **ND6** | Range-1 | Average distance | Range-2 |
| *D. punctatus-D. punctatus* | 0.0154 | 0.0154 | 0.0154 |
| *D. punctatus-D. punctatus_ws* | 0.0015 | 0.0100 | 0.0185 |
| *D. punctatus-D. tabulaeformis* | 0.0030 | 0.0092 | 0.0154 |
| *D. punctatus-D. spectabilis* | 0.0546 | 0.0562 | 0.0578 |
| **tRNA** | Range-1 | Average distance | Range-2 |
| *D. punctatus-D. punctatus* | 0.0034 | 0.0034 | 0.0034 |
| *D. punctatus-D. punctatus_ws* | 0.0014 | 0.0024 | 0.0034 |
| *D. punctatus-D. tabulaeformis* | 0.0014 | 0.0020 | 0.0027 |
| *D. punctatus-D. spectabilis* | 0.0139 | 0.0142 | 0.0146 |
| **rRNA** | Range-1 | Average distance | Range-2 |
| *D. punctatus-D. punctatus* | 0.0079 | 0.0079 | 0.0079 |
| *D. punctatus-D. punctatus_ws* | 0.0025 | 0.0056 | 0.0087 |
| *D. punctatus-D. tabulaeformis* | 0.0025 | 0.0068 | 0.0112 |
| *D. punctatus-D. spectabilis* | 0.0320 | 0.0333 | 0.0346 |
| **Noncoding** | Range-1 | Average distance | Range-2 |
| *D. punctatus-D. punctatus* | 0.0480 | 0.0480 | 0.0480 |
| *D. punctatus-D. punctatus_ws* | 0.0045 | 0.0234 | 0.0424 |
| *D. punctatus-D. tabulaeformis* | 0.0022 | 0.0236 | 0.0450 |
| *D. punctatus-D. spectabilis* | 0.1110 | 0.1121 | 0.1132 |
| **ATP8** | Range-1 | Average distance | Range-2 |
| *D. punctatus-D. punctatus* | 0.0064 | 0.0064 | 0.0064 |
| *D. punctatus-D. punctatus_ws* | 0.0000 | 0.0032 | 0.0064 |
| *D. punctatus-D. tabulaeformis* | 0.0000 | 0.0132 | 0.0267 |
| *D. punctatus-D. spectabilis* | 0.0501 | 0.0542 | 0.0582 |
| **COX2** | Range-1 | Average distance | Range-2 |
| *D. punctatus-D. punctatus* | 0.0226 | 0.0226 | 0.0226 |
| *D. punctatus-D. punctatus_ws* | 0.0089 | 0.0173 | 0.0272 |
| *D. punctatus-D. tabulaeformis* | 0.0059 | 0.0251 | 0.0399 |
| *D. punctatus-D. spectabilis* | 0.0498 | 0.0539 | 0.0580 |
| **COX3** | Range-1 | Average distance | Range-2 |
| *D. punctatus-D. punctatus* | 0.0168 | 0.0168 | 0.0168 |
| *D. punctatus-D. punctatus_ws* | 0.0077 | 0.0142 | 0.0221 |
| *D. punctatus-D. tabulaeformis* | 0.0051 | 0.0188 | 0.0287 |
| *D. punctatus-D. spectabilis* | 0.0496 | 0.0503 | 0.0511 |
| **Cytb** | Range-1 | Average distance | Range-2 |
| *D. punctatus-D. punctatus* | 0.0112 | 0.0112 | 0.0112 |
| *D. punctatus-D. punctatus_ws* | 0.0052 | 0.0082 | 0.0106 |
| *D. punctatus-D. tabulaeformis* | 0.0035 | 0.0134 | 0.0210 |
| *D. punctatus-D. spectabilis* | 0.0514 | 0.0528 | 0.0542 |
| **ND1** | Range-1 | Average distance | Range-2 |
| *D. punctatus-D. punctatus* | 0.0171 | 0.0171 | 0.0171 |
| *D. punctatus-D. punctatus_ws* | 0.0042 | 0.0123 | 0.0192 |
| *D. punctatus-D. tabulaeformis* | 0.0053 | 0.0199 | 0.0282 |
| *D. punctatus-D. spectabilis* | 0.0395 | 0.0395 | 0.0395 |
| **ND3** | Range-1 | Average distance | Range-2 |
| *D. punctatus-D. punctatus* | 0.0057 | 0.0057 | 0.0057 |
| *D. punctatus-D. punctatus_ws* | 0.0028 | 0.0043 | 0.0057 |
| *D. punctatus-D. tabulaeformis* | 0.0028 | 0.0065 | 0.0086 |
| *D. punctatus-D. spectabilis* | 0.0453 | 0.0470 | 0.0486 |
