# Supplementary material for: Mitochondrial phylogenomics and genetic relationships of closely related pine moth (Lasiocampidae: Dendrolimus) species in China, using whole mitochondrial genomes
Source: BMC Genomics. 2015 Jun 4;16(1):428. doi: 10.1186/s12864-015-1566-5 (PMC4455531; doi:10.1186/s12864-015-1566-5)
Supplement: Additional file 5: — (a). Annotation of the mitochondrial genome of D. spectabilis 02; (b). Annotation of the mitochondrial genome of D. spectabilis 13; (c). Annotation of the mitochondrial genome of D. punctatus 04; (d). Annotation of the mitochondrial genome of D. punctatus 05; (e). Annotation of the mitochondrial genome of D. punctatus wenshanensis 03; (f). Annotation of the mitochondrial genome of D. punctatus wenshanensis 06; (g). Annotation of the mitochondrial genome of D.tabulaeformis 06; (h). Annotation of the mitochondrial genome of D.tabulaeformis 38. [file 12864_2015_1566_MOESM5_ESM.docx]

Additional file 5 (a) Annotation of the mitochondrial genome of *D. spectabilis02*

| Gene | Direction | | Location | | Size | Anticodon | Start codon | Stop codon | Intergenic nucleotides |
| --- | --- | --- | --- | --- | --- | --- | --- | --- | --- |
| trnM | | F | 1 | 67 | 67 | CAT |  |  | 3 |
| trnI | | F | 71 | 134 | 64 | GAT |  |  | -3 |
| trnQ | | R | 132 | 200 | 69 | TTG |  |  | 58 |
| nd2 | | F | 259 | 1266 | 1008 |  | ATT | TAA | -2 |
| trnW | | F | 1265 | 1333 | 69 | TCA |  |  | -8 |
| trnC | | R | 1326 | 1391 | 66 | GCA |  |  | 0 |
| trnY | | R | 1392 | 1459 | 68 | GTA |  |  | 25 |
| cox1 | | F | 1485 | 3015 | 1531 |  | CGA | T | 0 |
| trnL(UUR) | | F | 3016 | 3082 | 67 | TAA |  |  | 0 |
| cox2 | | F | 3083 | 3764 | 682 |  | ATA | T | 0 |
| trnK | | F | 3765 | 3835 | 71 | CTT |  |  | 3 |
| trnD | | F | 3839 | 3906 | 68 | GTC |  |  | 0 |
| atp8 | | F | 3907 | 4065 | 159 |  | ATC | TAA | -7 |
| atp6 | | F | 4059 | 4736 | 678 |  | ATG | TAA | 11 |
| cox3 | | F | 4748 | 5536 | 789 |  | ATG | TAA | 2 |
| trnG | | F | 5539 | 5604 | 66 | TCC |  |  | 0 |
| nd3 | | F | 5605 | 5957 | 353 |  | ATC | TA | 0 |
| trnA | | F | 5958 | 6024 | 67 | TGC |  |  | 20 |
| trnR | | F | 6045 | 6108 | 64 | TCG |  |  | 4 |
| trnN | | F | 6113 | 6178 | 66 | GTT |  |  | 18 |
| trnS(AGN) | | F | 6197 | 6264 | 68 | GCT |  |  | -1 |
| trnE | | F | 6264 | 6328 | 65 | TTC |  |  | 8 |
| trnF | | R | 6337 | 6402 | 66 | GAA |  |  | 3 |
| nd5 | | R | 6406 | 8148 | 1743 |  | ATT | TAA | 0 |
| trnH | | R | 8149 | 8216 | 68 | GTG |  |  | 0 |
| nd4 | | R | 8217 | 9555 | 1339 |  | ATG | T | 23 |
| nd4l | | R | 9579 | 9872 | 294 |  | ATG | TAA | 7 |
| trnT | | F | 9880 | 9943 | 64 | TGT |  |  | 0 |
| trnP | | R | 9944 | 10008 | 65 | TGG |  |  | 8 |
| nd6 | | F | 10017 | 10547 | 531 |  | ATA | TAA | 4 |
| cytb | | F | 10552 | 11700 | 1149 |  | ATG | TAA | 3 |
| trnS(UCN) | | F | 11704 | 11769 | 66 | TGA |  |  | -1 |
| nd1 | | R | 11769 | 12722 | 954 |  | ATG | TAA | 1 |
| trnL(CUN) | | R | 12724 | 12791 | 68 | TAG |  |  | 0 |
| rrnL | | R | 12792 | 14245 | 1454 |  |  |  | 0 |
| trnV | | R | 14246 | 14311 | 66 | TAC |  |  | 0 |
| rrnS | | R | 14312 | 15092 | 781 |  |  |  | 0 |
| AT region | | F | 15093 | 15412 | 320 |  |  |  | 0 |

Additional file 5 (b) Annotation of the mitochondrial genome of *D. spectabilis13*

| Gene | Direction | | Location | | Size | Anticodon | Start codon | Stop codon | Intergenic nucleotides |
| --- | --- | --- | --- | --- | --- | --- | --- | --- | --- |
| trnM | F | 1 | | 67 | 67 | CAT |  |  | 3 |
| trnI | F | 71 | | 134 | 64 | GAT |  |  | -3 |
| trnQ | R | 132 | | 200 | 69 | TTG |  |  | 58 |
| nd2 | F | 259 | | 1266 | 1008 |  | ATT | TAA | -2 |
| trnW | F | 1265 | | 1333 | 69 | TCA |  |  | -8 |
| trnC | R | 1326 | | 1391 | 66 | GCA |  |  | 0 |
| trnY | R | 1392 | | 1459 | 68 | GTA |  |  | 25 |
| cox1 | F | 1485 | | 3015 | 1531 |  | CGA | T | 0 |
| trnL(UUR) | F | 3016 | | 3082 | 67 | TAA |  |  | 0 |
| cox2 | F | 3083 | | 3764 | 682 |  | ATA | T | 0 |
| trnK | F | 3765 | | 3835 | 71 | CTT |  |  | 3 |
| trnD | F | 3839 | | 3906 | 68 | GTC |  |  | 0 |
| atp8 | F | 3907 | | 4065 | 159 |  | ATC | TAA | -7 |
| atp6 | F | 4059 | | 4736 | 678 |  | ATG | TAA | 11 |
| cox3 | F | 4748 | | 5536 | 789 |  | ATG | TAA | 2 |
| trnG | F | 5539 | | 5604 | 66 | TCC |  |  | 0 |
| nd3 | F | 5605 | | 5957 | 353 |  | ATC | TA | 0 |
| trnA | F | 5958 | | 6024 | 67 | TGC |  |  | 20 |
| trnR | F | 6045 | | 6108 | 64 | TCG |  |  | 4 |
| trnN | F | 6113 | | 6178 | 66 | GTT |  |  | 18 |
| trnS(AGN) | F | 6197 | | 6264 | 68 | GCT |  |  | -1 |
| trnE | F | 6264 | | 6328 | 65 | TTC |  |  | 8 |
| trnF | R | 6337 | | 6402 | 66 | GAA |  |  | 3 |
| nd5 | R | 6406 | | 8148 | 1743 |  | ATT | TAA | 0 |
| trnH | R | 8149 | | 8216 | 68 | GTG |  |  | 0 |
| nd4 | R | 8217 | | 9555 | 1339 |  | ATG | T | 23 |
| nd4l | R | 9579 | | 9872 | 294 |  | ATG | TAA | 7 |
| trnT | F | 9880 | | 9943 | 64 | TGT |  |  | 0 |
| trnP | R | 9944 | | 10008 | 65 | TGG |  |  | 8 |
| nd6 | F | 10017 | | 10547 | 531 |  | ATA | TAA | 4 |
| cytb | F | 10552 | | 11700 | 1149 |  | ATG | TAA | 3 |
| trnS(UCN) | F | 11704 | | 11769 | 66 | TGA |  |  | -1 |
| nd1 | R | 11769 | | 12722 | 954 |  | ATG | TAA | 1 |
| trnL(CUN) | R | 12724 | | 12791 | 68 | TAG |  |  | 0 |
| rrnL | R | 12792 | | 14245 | 1454 |  |  |  | 0 |
| trnV | R | 14246 | | 14311 | 66 | TAC |  |  | 0 |
| rrnS | R | 14312 | | 15090 | 779 |  |  |  | 0 |
| AT region | F | 15091 | | 15410 | 320 |  |  |  | 0 |

Additional file 5 (c) Annotation of the mitochondrial genome of *D. punctatus 04*

| Gene | Direction | Location | | Size | Anticodon | Start codon | Stop codon | Intergenic nucleotides |
| --- | --- | --- | --- | --- | --- | --- | --- | --- |
| trnM | F | 1 | 67 | 67 | CAT |  |  | 3 |
| trnI | F | 71 | 134 | 64 | GAT |  |  | -3 |
| trnQ | R | 132 | 200 | 69 | TTG |  |  | 58 |
| nd2 | F | 259 | 1266 | 1008 |  | ATT | TAA | -2 |
| trnW | F | 1265 | 1333 | 69 | TCA |  |  | -8 |
| trnC | R | 1326 | 1391 | 66 | GCA |  |  | 0 |
| trnY | R | 1392 | 1459 | 68 | GTA |  |  | 27 |
| cox1 | F | 1487 | 3017 | 1531 |  | CGA | T | 0 |
| trnL(UUR) | F | 3018 | 3084 | 67 | TAA |  |  | 0 |
| cox2 | F | 3085 | 3766 | 682 |  | ATA | T | 0 |
| trnK | F | 3767 | 3837 | 71 | CTT |  |  | 3 |
| trnD | F | 3841 | 3908 | 68 | GTC |  |  | 0 |
| atp8 | F | 3909 | 4070 | 162 |  | ATT | TAA | -7 |
| atp6 | F | 4064 | 4741 | 678 |  | ATG | TAA | 15 |
| cox3 | F | 4757 | 5545 | 789 |  | ATG | TAA | 2 |
| trnG | F | 5548 | 5613 | 66 | TCC |  |  | 0 |
| nd3 | F | 5614 | 5966 | 353 |  | ATG | TA | 0 |
| trnA | F | 5967 | 6033 | 67 | TGC |  |  | 15 |
| trnR | F | 6049 | 6112 | 64 | TCG |  |  | 4 |
| trnN | F | 6117 | 6182 | 66 | GTT |  |  | 11 |
| trnS(AGN) | F | 6194 | 6261 | 68 | GCT |  |  | -1 |
| trnE | F | 6261 | 6326 | 66 | TTC |  |  | 4 |
| trnF | R | 6331 | 6396 | 66 | GAA |  |  | 2 |
| nd5 | R | 6399 | 8141 | 1743 |  | ATT | TAA | 0 |
| trnH | R | 8142 | 8209 | 68 | GTG |  |  | 0 |
| nd4 | R | 8210 | 9548 | 1339 |  | ATG | T | 24 |
| nd4l | R | 9573 | 9866 | 294 |  | ATG | TAA | 7 |
| trnT | F | 9874 | 9937 | 64 | TGT |  |  | 0 |
| trnP | R | 9938 | 10002 | 65 | TGG |  |  | 8 |
| nd6 | F | 10011 | 10541 | 531 |  | ATA | TAA | 4 |
| cytb | F | 10546 | 11694 | 1149 |  | ATG | TAA | 3 |
| trnS(UCN) | F | 11698 | 11764 | 67 | TGA |  |  | -1 |
| nd1 | R | 11764 | 12717 | 954 |  | ATG | TAA | 1 |
| trnL(CUN) | R | 12719 | 12786 | 68 | TAG |  |  | 0 |
| rrnL | R | 12787 | 14247 | 1461 |  |  |  | 0 |
| trnV | R | 14248 | 14312 | 65 | TAC |  |  | 0 |
| rrnS | R | 14313 | 15091 | 779 |  |  |  | 0 |
| AT region | F | 15092 | 15411 | 320 |  |  |  | 0 |

Additional file 5 (d) Annotation of the mitochondrial genome of *D. punctatus05*

| Gene | Direction | | Location | | Size | Anticodon | Start codon | Stop codon | Intergenic nucleotides |
| --- | --- | --- | --- | --- | --- | --- | --- | --- | --- |
| trnM | | F | 1 | 67 | 67 | CAT |  |  | 3 |
| trnI | | F | 71 | 134 | 64 | GAT |  |  | -3 |
| trnQ | | R | 132 | 200 | 69 | TTG |  |  | 58 |
| nd2 | | F | 259 | 1266 | 1008 |  | ATT | TAA | -2 |
| trnW | | F | 1265 | 1333 | 69 | TCA |  |  | -8 |
| trnC | | R | 1326 | 1391 | 66 | GCA |  |  | 0 |
| trnY | | R | 1392 | 1459 | 68 | GTA |  |  | 34 |
| cox1 | | F | 1494 | 3024 | 1531 |  | CGA | T | 0 |
| trnL(UUR) | | F | 3025 | 3091 | 67 | TAA |  |  | 0 |
| cox2 | | F | 3092 | 3773 | 682 |  | ATA | T | 0 |
| trnK | | F | 3774 | 3844 | 71 | CTT |  |  | 3 |
| trnD | | F | 3848 | 3915 | 68 | GTC |  |  | 0 |
| atp8 | | F | 3916 | 4077 | 162 |  | ATT | TAA | -7 |
| atp6 | | F | 4071 | 4748 | 678 |  | ATG | TAA | 14 |
| cox3 | | F | 4763 | 5551 | 789 |  | ATG | TAA | 2 |
| trnG | | F | 5554 | 5619 | 66 | TCC |  |  | 0 |
| nd3 | | F | 5620 | 5972 | 353 |  | ATA | TA | 0 |
| trnA | | F | 5973 | 6039 | 67 | TGC |  |  | 15 |
| trnR | | F | 6055 | 6118 | 64 | TCG |  |  | 4 |
| trnN | | F | 6123 | 6188 | 66 | GTT |  |  | 13 |
| trnS(AGN) | | F | 6202 | 6269 | 68 | GCT |  |  | -1 |
| trnE | | F | 6269 | 6333 | 65 | TTC |  |  | 4 |
| trnF | | R | 6338 | 6403 | 66 | GAA |  |  | 3 |
| nd5 | | R | 6407 | 8149 | 1743 |  | ATT | TAA | 0 |
| trnH | | R | 8150 | 8217 | 68 | GTG |  |  | 0 |
| nd4 | | R | 8218 | 9556 | 1339 |  | ATG | T | 19 |
| nd4l | | R | 9576 | 9869 | 294 |  | ATG | TAA | 7 |
| trnT | | F | 9877 | 9941 | 65 | TGT |  |  | 0 |
| trnP | | R | 9942 | 10006 | 65 | TGG |  |  | 8 |
| nd6 | | F | 10015 | 10545 | 531 |  | ATA | TAA | 4 |
| cytb | | F | 10550 | 11698 | 1149 |  | ATG | TAA | 3 |
| trnS(UCN) | | F | 11702 | 11768 | 67 | TGA |  |  | -1 |
| nd1 | | R | 11768 | 12721 | 954 |  | ATG | TAA | 1 |
| trnL(CUN) | | R | 12723 | 12790 | 68 | TAG |  |  | 0 |
| rrnL | | R | 12791 | 14242 | 1452 |  |  |  | 0 |
| trnV | | R | 14243 | 14307 | 65 | TAC |  |  | 0 |
| rrnS | | R | 14308 | 15087 | 780 |  |  |  | 0 |
| AT region | | F | 15088 | 15407 | 320 |  |  |  | 0 |

Additional file 5 (e) Annotation of the mitochondrial genome of *D. punctatus wenshanensis03*

| Gene | Direction | Location | | Size | Start codon | Stop codon | Anticodon | Intergenic nucleotides |
| --- | --- | --- | --- | --- | --- | --- | --- | --- |
| trnM | F | 1 | 67 | 67 |  |  | CAT | 3 |
| trnI | F | 71 | 134 | 64 |  |  | GAT | -3 |
| trnQ | R | 132 | 200 | 69 |  |  | TTG | 58 |
| nd2 | F | 259 | 1266 | 1008 | ATT | TAA |  | -2 |
| trnW | F | 1265 | 1333 | 69 |  |  | TCA | -8 |
| trnC | R | 1326 | 1391 | 66 |  |  | GCA | 0 |
| trnY | R | 1392 | 1459 | 68 |  |  | GTA | 34 |
| cox1 | F | 1494 | 3024 | 1531 | CGA | T |  | 0 |
| trnL(UUR) | F | 3025 | 3091 | 67 |  |  | TAA | 0 |
| cox2 | F | 3092 | 3773 | 682 | ATA | T |  | 0 |
| trnK | F | 3774 | 3844 | 71 |  |  | CTT | 3 |
| trnD | F | 3848 | 3915 | 68 |  |  | GTC | 0 |
| atp8 | F | 3916 | 4077 | 162 | ATT | TAA |  | -7 |
| atp6 | F | 4071 | 4748 | 678 | ATG | TAA |  | 15 |
| cox3 | F | 4764 | 5552 | 789 | ATG | TAA |  | 2 |
| trnG | F | 5555 | 5620 | 66 |  |  | TCC | 0 |
| nd3 | F | 5621 | 5973 | 353 | ATG | TA |  | 0 |
| trnA | F | 5974 | 6040 | 67 |  |  | TGC | 15 |
| trnR | F | 6056 | 6119 | 64 |  |  | TCG | 4 |
| trnN | F | 6124 | 6189 | 66 |  |  | GTT | 11 |
| trnS(AGN) | F | 6201 | 6268 | 68 |  |  | GCT | -1 |
| trnE | F | 6268 | 6333 | 66 |  |  | TTC | 4 |
| trnF | R | 6338 | 6403 | 66 |  |  | GAA | 2 |
| nd5 | R | 6406 | 8148 | 1743 | ATT | TAA |  | 0 |
| trnH | R | 8149 | 8216 | 68 |  |  | GTG | 0 |
| nd4 | R | 8217 | 9555 | 1339 | ATG | T |  | 24 |
| nd4l | R | 9580 | 9873 | 294 | ATG | TAA |  | 7 |
| trnT | F | 9881 | 9944 | 64 |  |  | TGT | 0 |
| trnP | R | 9945 | 10009 | 65 |  |  | TGG | 8 |
| nd6 | F | 10018 | 10548 | 531 | ATA | TAA |  | 4 |
| cytb | F | 10553 | 11701 | 1149 | ATG | TAA |  | 3 |
| trnS(UCN) | F | 11705 | 11771 | 67 |  |  | TGA | -1 |
| nd1 | R | 11771 | 12724 | 954 | ATG | TAA |  | 1 |
| trnL(CUN) | R | 12726 | 12793 | 68 |  |  | TAG | 0 |
| rrnL | R | 12794 | 14255 | 1462 |  |  |  | 0 |
| trnV | R | 14256 | 14320 | 65 |  |  | TAC | 0 |
| rrnS | R | 14321 | 15099 | 779 |  |  |  | 0 |
| AT region | F | 15100 | 15419 | 320 |  |  |  | 0 |

Additional file 5 (f) Annotation of the mitochondrial genome of *D. punctatus wenshanensis06*

| Gene | Direction | Location | | Size | Anticodon | Start codon | Stop codon | Intergenic nucleotides |
| --- | --- | --- | --- | --- | --- | --- | --- | --- |
| trnM | F | 1 | 67 | 67 | CAT |  |  | 3 |
| trnI | F | 71 | 134 | 64 | GAT |  |  | -3 |
| trnQ | R | 132 | 200 | 69 | TTG |  |  | 58 |
| nd2 | F | 259 | 1266 | 1008 |  | ATT | TAA | -2 |
| trnW | F | 1265 | 1333 | 69 | TCA |  |  | -8 |
| trnC | R | 1326 | 1391 | 66 | GCA |  |  | 0 |
| trnY | R | 1392 | 1459 | 68 | GTA |  |  | 34 |
| cox1 | F | 1494 | 3024 | 1531 |  | CGA | T | 0 |
| trnL(UUR) | F | 3025 | 3091 | 67 | TAA |  |  | 0 |
| cox2 | F | 3092 | 3773 | 682 |  | ATA | T | 0 |
| trnK | F | 3774 | 3844 | 71 | CTT |  |  | 3 |
| trnD | F | 3848 | 3915 | 68 | GTC |  |  | 0 |
| atp8 | F | 3916 | 4077 | 162 |  | ATT | TAA | -7 |
| atp6 | F | 4071 | 4748 | 678 |  | ATG | TAA | 15 |
| cox3 | F | 4764 | 5552 | 789 |  | ATG | TAA | 2 |
| trnG | F | 5555 | 5620 | 66 | TCC |  |  | 0 |
| nd3 | F | 5621 | 5973 | 353 |  | ATG | TA | 0 |
| trnA | F | 5974 | 6040 | 67 | TGC |  |  | 15 |
| trnR | F | 6056 | 6119 | 64 | TCG |  |  | 4 |
| trnN | F | 6124 | 6189 | 66 | GTT |  |  | 11 |
| trnS(AGN) | F | 6201 | 6268 | 68 | GCT |  |  | -1 |
| trnE | F | 6268 | 6332 | 65 | TTC |  |  | 4 |
| trnF | R | 6337 | 6402 | 66 | GAA |  |  | 2 |
| nd5 | R | 6405 | 8147 | 1743 |  | ATT | TAA | 0 |
| trnH | R | 8148 | 8215 | 68 | GTG |  |  | 0 |
| nd4 | R | 8216 | 9554 | 1339 |  | ATG | T | 24 |
| nd4l | R | 9579 | 9872 | 294 |  | ATG | TAA | 7 |
| trnT | F | 9880 | 9943 | 64 | TGT |  |  | 0 |
| trnP | R | 9944 | 10008 | 65 | TGG |  |  | 8 |
| nd6 | F | 10017 | 10547 | 531 |  | ATA | TAA | 4 |
| cytb | F | 10552 | 11700 | 1149 |  | ATG | TAA | 3 |
| trnS(UCN) | F | 11704 | 11770 | 67 | TGA |  |  | -1 |
| nd1 | R | 11770 | 12723 | 954 |  | ATG | TAA | 1 |
| trnL(CUN) | R | 12725 | 12792 | 68 | TAG |  |  | 0 |
| rrnL | R | 12793 | 14254 | 1462 |  |  |  | 0 |
| trnV | R | 14255 | 14319 | 65 | TAC |  |  | 0 |
| rrnS | R | 14320 | 15098 | 779 |  |  |  | 0 |
| AT region | F | 15099 | 15418 | 320 |  |  |  | 0 |

Additional file 5 (g) Annotation of the mitochondrial genome of *D.tabulaeformis06*

| Gene | Direction | Location | | Size | Anticodon | Start codon | Stop codon | Intergenic nucleotides |
| --- | --- | --- | --- | --- | --- | --- | --- | --- |
| trnM | F | 1 | 67 | 67 | CAT |  |  | 3 |
| trnI | F | 71 | 134 | 64 | GAT |  |  | -3 |
| trnQ | R | 132 | 200 | 69 | TTG |  |  | 58 |
| nd2 | F | 259 | 1266 | 1008 |  | ATT | TAA | -2 |
| trnW | F | 1265 | 1333 | 69 | TCA |  |  | -8 |
| trnC | R | 1326 | 1391 | 66 | GCA |  |  | 0 |
| trnY | R | 1392 | 1459 | 68 | GTA |  |  | 34 |
| cox1 | F | 1494 | 3024 | 1531 |  | CGA | T | 0 |
| trnL(UUR) | F | 3025 | 3091 | 67 | TAA |  |  | 0 |
| cox2 | F | 3092 | 3773 | 682 |  | ATA | T | 0 |
| trnK | F | 3774 | 3844 | 71 | CTT |  |  | 3 |
| trnD | F | 3848 | 3915 | 68 | GTC |  |  | 0 |
| atp8 | F | 3916 | 4074 | 159 |  | ATT | TAA | -7 |
| atp6 | F | 4068 | 4745 | 678 |  | ATG | TAA | 15 |
| cox3 | F | 4761 | 5549 | 789 |  | ATG | TAA | 2 |
| trnG | F | 5552 | 5617 | 66 | TCC |  |  | 0 |
| nd3 | F | 5618 | 5970 | 353 |  | ATG | TA | 0 |
| trnA | F | 5971 | 6037 | 67 | TGC |  |  | 15 |
| trnR | F | 6053 | 6116 | 64 | TCG |  |  | 4 |
| trnN | F | 6121 | 6186 | 66 | GTT |  |  | 11 |
| trnS(AGN) | F | 6198 | 6265 | 68 | GCT |  |  | -1 |
| trnE | F | 6265 | 6329 | 65 | TTC |  |  | 4 |
| trnF | R | 6334 | 6399 | 66 | GAA |  |  | 2 |
| nd5 | R | 6402 | 8144 | 1743 |  | ATT | TAA | 0 |
| trnH | R | 8145 | 8212 | 68 | GTG |  |  | 0 |
| nd4 | R | 8213 | 9551 | 1339 |  | ATG | T | 24 |
| nd4l | R | 9576 | 9869 | 294 |  | ATG | TAA | 7 |
| trnT | F | 9877 | 9940 | 64 | TGT |  |  | 0 |
| trnP | R | 9941 | 10005 | 65 | TGG |  |  | 8 |
| nd6 | F | 10014 | 10544 | 531 |  | ATA | TAA | 4 |
| cytb | F | 10549 | 11697 | 1149 |  | ATG | TAA | 3 |
| trnS(UCN) | F | 11701 | 11767 | 67 | TGA |  |  | -1 |
| nd1 | R | 11767 | 12720 | 954 |  | ATG | TAA | 1 |
| trnL(CUN) | R | 12722 | 12789 | 68 | TAG |  |  | 0 |
| rrnL | R | 12790 | 14248 | 1459 |  |  |  | 0 |
| trnV | R | 14249 | 14313 | 65 | TAC |  |  | 0 |
| rrnS | R | 14314 | 15091 | 778 |  |  |  | 0 |
| AT region | F | 15092 | 15411 | 320 |  |  |  | 0 |

Additional file 5 (h) Annotation of the mitochondrial genome of *D.tabulaeformis38*

| Gene | Direction | Location | | Size | Anticodon | Start codon | Stop codon | Intergenic nucleotides |
| --- | --- | --- | --- | --- | --- | --- | --- | --- |
| trnM | F | 1 | 67 | 67 | CAT |  |  | 3 |
| trnI | F | 71 | 134 | 64 | GAT |  |  | -3 |
| trnQ | R | 132 | 200 | 69 | TTG |  |  | 58 |
| nd2 | F | 259 | 1266 | 1008 |  | ATT | TAA | -2 |
| trnW | F | 1265 | 1333 | 69 | TCA |  |  | -8 |
| trnC | R | 1326 | 1391 | 66 | GCA |  |  | 0 |
| trnY | R | 1392 | 1459 | 68 | GTA |  |  | 34 |
| cox1 | F | 1494 | 3024 | 1531 |  | CGA | T | 0 |
| trnL(UUR) | F | 3025 | 3091 | 67 | TAA |  |  | 0 |
| cox2 | F | 3092 | 3773 | 682 |  | ATA | T | 0 |
| trnK | F | 3774 | 3844 | 71 | CTT |  |  | 3 |
| trnD | F | 3848 | 3915 | 68 | GTC |  |  | 0 |
| atp8 | F | 3916 | 4074 | 159 |  | ATC | TAA | -7 |
| atp6 | F | 4068 | 4745 | 678 |  | ATG | TAA | 15 |
| cox3 | F | 4761 | 5549 | 789 |  | ATG | TAA | 2 |
| trnG | F | 5552 | 5617 | 66 | TCC |  |  | 0 |
| nd3 | F | 5618 | 5970 | 353 |  | ATT | TA | 0 |
| trnA | F | 5971 | 6037 | 67 | TGC |  |  | 15 |
| trnR | F | 6053 | 6116 | 64 | TCG |  |  | 4 |
| trnN | F | 6121 | 6186 | 66 | GTT |  |  | 11 |
| trnS(AGN) | F | 6198 | 6265 | 68 | GCT |  |  | -1 |
| trnE | F | 6265 | 6329 | 65 | TTC |  |  | 4 |
| trnF | R | 6334 | 6399 | 66 | GAA |  |  | 2 |
| nd5 | R | 6402 | 8144 | 1743 |  | ATT | TAA | 0 |
| trnH | R | 8145 | 8212 | 68 | GTG |  |  | 0 |
| nd4 | R | 8213 | 9551 | 1339 |  | ATG | T | 24 |
| nd4l | R | 9576 | 9869 | 294 |  | ATG | TAA | 7 |
| trnT | F | 9877 | 9940 | 64 | TGT |  |  | 0 |
| trnP | R | 9941 | 10005 | 65 | TGG |  |  | 8 |
| nd6 | F | 10014 | 10544 | 531 |  | ATA | TAA | 4 |
| cytb | F | 10549 | 11697 | 1149 |  | ATG | TAA | 3 |
| trnS(UCN) | F | 11701 | 11767 | 67 | TGA |  |  | -1 |
| nd1 | R | 11767 | 12720 | 954 |  | ATG | TAA | 1 |
| trnL(CUN) | R | 12722 | 12789 | 68 | TAG |  |  | 0 |
| rrnL | R | 12790 | 14245 | 1456 |  |  |  | 0 |
| trnV | R | 14246 | 14310 | 65 | TAC |  |  | 0 |
| rrnS | R | 14311 | 15089 | 779 |  |  |  | 0 |
| AT region | F | 15090 | 15409 | 320 |  |  |  | 0 |
