# Supplementary material for: Mitochondrial phylogenomics and genetic relationships of closely related pine moth (Lasiocampidae: Dendrolimus) species in China, using whole mitochondrial genomes
Source: BMC Genomics. 2015 Jun 4;16(1):428. doi: 10.1186/s12864-015-1566-5 (PMC4455531; doi:10.1186/s12864-015-1566-5)
Supplement: Additional file 6: — Start codons and stop codons of the eight sequenced individuals. [file 12864_2015_1566_MOESM6_ESM.docx]

Additional file 6 Start codons and stop codons of the eight sequenced individuals.

| Sample | nd2 | cox1 | cox2 | atp8 | atp6 | cox3 | nd3 | nd5 | nd4 | nd4l | nd6 | cob | nd1 |
| --- | --- | --- | --- | --- | --- | --- | --- | --- | --- | --- | --- | --- | --- |
| *D. spectabilis02* | ATT/TAA | CGA/T | ATA/T | ATC/TAA | ATG/TAA | ATG/TAA | ATC/TA | ATT/TAA | ATG/T | ATG/TAA | ATA/TAA | ATG/TAA | ATG/TAA |
| *D. spectabilis13* | ATT/TAA | CGA/T | ATA/T | ATC/TAA | ATG/TAA | ATG/TAA | ATC/TA | ATT/TAA | ATG/T | ATG/TAA | ATA/TAA | ATG/TAA | ATG/TAA |
| *D. tabulaeformis06* | ATT/TAA | CGA/T | ATA/T | ATT/TAA | ATG/TAA | ATG/TAA | ATG/TA | ATT/TAA | ATG/T | ATG/TAA | ATA/TAA | ATG/TAA | ATG/TAA |
| *D. tabulaeformis38* | ATT/TAA | CGA/T | ATA/T | ATC/TAA | ATG/TAA | ATG/TAA | ATT/TA | ATT/TAA | ATG/T | ATG/TAA | ATA/TAA | ATG/TAA | ATG/TAA |
| *D. punctatus04* | ATT/TAA | CGA/T | ATA/T | ATT/TAA | ATG/TAA | ATG/TAA | ATG/TA | ATT/TAA | ATG/T | ATG/TAA | ATA/TAA | ATG/TAA | ATG/TAA |
| *D. punctatus05* | ATT/TAA | CGA/T | ATA/T | ATT/TAA | ATG/TAA | ATG/TAA | ATA/TA | ATT/TAA | ATG/T | ATG/TAA | ATA/TAA | ATG/TAA | ATG/TAA |
| *D. punctatus_ws03* | ATT/TAA | CGA/T | ATA/T | ATT/TAA | ATG/TAA | ATG/TAA | ATG/TA | ATT/TAA | ATG/T | ATG/TAA | ATA/TAA | ATG/TAA | ATG/TAA |
| *D. punctatus_ws06* | ATT/TAA | CGA/T | ATA/T | ATT/TAA | ATG/TAA | ATG/TAA | ATG/TA | ATT/TAA | ATG/T | ATG/TAA | ATA/TAA | ATG/TAA | ATG/TAA |
