# Supplementary material for: Mitochondrial phylogenomics and genetic relationships of closely related pine moth (Lasiocampidae: Dendrolimus) species in China, using whole mitochondrial genomes
Source: BMC Genomics. 2015 Jun 4;16(1):428. doi: 10.1186/s12864-015-1566-5 (PMC4455531; doi:10.1186/s12864-015-1566-5)
Supplement: Additional file 7: — Numbers of mismatched base pairs in the eight sequenced genomes. [file 12864_2015_1566_MOESM7_ESM.docx]

Additional file 7 Numbers of mismatched base pairs in the eight sequenced genomes.

| Sample | Total | Different mismatches | Identical mismatches |
| --- | --- | --- | --- |
| *D. spectabilis02* | 19 | trnM:1G-U(DHU); trnQ:1G-U(anti); trnL(UUN):1U-U(aaa); | trnA:1U-U(aaa),1G-U(aaa); |
| *D. spectabilis13* | 19 | trnM:1G-U(DHU); trnQ:1G-U(anti); trnL(UUN):1U-U(aaa); | trnC:1G-U(aaa); trnG:1G-U(DHU); |
| *D. tabulaeformis06* | 20 | trnM:1G-U(DHU); trnQ:1G-U(anti); trnL(UUN):1U-U(aaa),1G-U(DHU); | trnH:1G-U(anti); trnP:1G-U(DHU); |
| *D. tabulaeformis38* | 21 | trnM:1G-U(DHU); trnQ:1G-U(anti); trnL(UUN):1U-U(aaa),1G-U(DHU); | trnL(CNU):1G-U(aaa),1G-U(DHU); |
| *D. punctatus04* | 21 | trnM:1G-U(DHU); trnQ:1G-U(anti); trnL(UUN):1U-U(aaa),1G-U(DHU); | trnF:1G-U(DHU),1G-U(DHU); |
| *D. punctatus05* | 19 | trnM:1G-U(DHU); trnQ:1G-U(anti); trnL(UUN):1U-U(aaa),1G-U(DHU); trnT:1G-U(aaa); | trnS(UCN):1U-U(anti); trnV:1G-U(DHU); |
| *D. punctatus_ws03* | 20 | trnM:1G-U(DHU),1G-U(anti); trnQ:1G-U(anti); trnL(UUN):1U-U(aaa),1G-U(DHU); | trnS(AGN):1C-A(anti),1U-G(TψC); |
| *D. punctatus_ws06* | 20 | trnM:1G-U(DHU); trnL(UUN):1U-U(aaa),1G-U(DHU); | trnW:1U-G(aaa),1U-G(anti); |
