# Supplementary material for: Mitochondrial phylogenomics and genetic relationships of closely related pine moth (Lasiocampidae: Dendrolimus) species in China, using whole mitochondrial genomes
Source: BMC Genomics. 2015 Jun 4;16(1):428. doi: 10.1186/s12864-015-1566-5 (PMC4455531; doi:10.1186/s12864-015-1566-5)
Supplement: Additional file 8: — Gene content of our eight sequenced specimens and other published Bombycoidea mitochondrial genomes. [file 12864_2015_1566_MOESM8_ESM.docx]

Additional file 8 Gene content of our eight sequenced specimens and other published Bombycoidea mitochondrial genomes.

| Sample | Size(bp) | A(％) | C(％) | G(％) | T(％) | A+T(％) | C+G(％) | AT-Skew | GC-Skew |
| --- | --- | --- | --- | --- | --- | --- | --- | --- | --- |
| Whole genome |  |  |  |  |  |  |  |  |  |
| *Bombyx mandarina* | 15928 | 43.1 | 11.1 | 7.2 | 38.6 | 81.7 | 18.3 | 0.05508 | -0.213115 |
| *Bombyx mori* | 15643 | 43 | 11.4 | 7.3 | 38.3 | 81.3 | 18.7 | 0.057811 | -0.219251 |
| *Actias selene* | 15236 | 38.5 | 13 | 8.1 | 40.4 | 78.9 | 21.1 | -0.024081 | -0.232227 |
| *Rondotia menciana* | 15301 | 41.4 | 13.3 | 7.8 | 37.4 | 78.8 | 21.1 | 0.050761 | -0.260664 |
| *Attacus atlas* | 15282 | 39.8 | 12.8 | 7.9 | 39.5 | 79.3 | 20.7 | 0.003783 | -0.236715 |
| *Samia cynthia ricini* | 15384 | 39.6 | 12.4 | 7.8 | 40.1 | 79.7 | 20.2 | -0.006274 | -0.227723 |
| *Antheraea yamamai* | 15338 | 39.3 | 12 | 7.7 | 41 | 80.3 | 19.7 | -0.021171 | -0.218274 |
| *Eriogyna pyretorum* | 15327 | 39.2 | 11.6 | 7.6 | 41.6 | 80.8 | 19.2 | -0.029703 | -0.208333 |
| *Saturnia boisduvalii* | 15360 | 39.3 | 11.8 | 7.6 | 41.3 | 80.6 | 19.4 | -0.024814 | -0.216495 |
| *Manduca sexta* | 15516 | 40.7 | 10.8 | 7.5 | 41.1 | 81.8 | 18.3 | -0.00489 | -0.180328 |
| *Antheraea pernyi* | 15566 | 39.2 | 12.1 | 7.8 | 40.9 | 80.1 | 19.9 | -0.021223 | -0.21608 |
| *Sphinx morio* | 15299 | 40.6 | 11.3 | 7.6 | 40.5 | 81.1 | 18.9 | 0.001233 | -0.195767 |
| *D. punctatus04* | 15411 | 40.9 | 12.5 | 8 | 38.6 | 79.5 | 20.5 | 0.028931 | -0.219512 |
| *D. punctatus05* | 15407 | 40.9 | 12.6 | 8 | 38.5 | 79.4 | 20.6 | 0.030227 | -0.223301 |
| *D. spectabilis02* | 15412 | 40.7 | 12.6 | 8 | 38.6 | 79.3 | 20.6 | 0.026482 | -0.223301 |
| *D. spectabilis13* | 15410 | 40.8 | 12.6 | 8 | 38.6 | 79.4 | 20.6 | 0.027708 | -0.223301 |
| *D. tabulaeformis06* | 15411 | 40.9 | 12.5 | 8 | 38.6 | 79.5 | 20.5 | 0.028931 | -0.219512 |
| *D. tabulaeformis38* | 15409 | 40.8 | 12.6 | 8 | 38.6 | 79.4 | 20.6 | 0.027708 | -0.223301 |
| *D. punctatus_ws03* | 15419 | 40.9 | 12.6 | 8 | 38.5 | 79.4 | 20.6 | 0.030227 | -0.223301 |
| *D. punctatus_ws06* | 15418 | 40.9 | 12.5 | 8 | 38.5 | 79.4 | 20.5 | 0.030227 | -0.219512 |
| PCGs |  |  |  |  |  |  | 0 |  |  |
| *Bombyx mandarina* | 11166 | 34.4 | 9.9 | 10.6 | 45.2 | 79.6 | 20.5 | -0.135678 | 0.0341463 |
| *Bombyx mori* | 11142 | 34.4 | 9.9 | 10.5 | 45.1 | 79.5 | 20.4 | -0.134591 | 0.0294118 |
| *Actias selene* | 11184 | 31.9 | 11.6 | 11.1 | 45.4 | 77.3 | 22.7 | -0.174644 | -0.022026 |
| *Rondotia menciana* | 11190 | 33.2 | 11.5 | 11.5 | 43.8 | 77 | 23 | -0.137662 | 0 |
| *Attacus atlas* | 11181 | 32.8 | 11.1 | 11.2 | 44.9 | 77.7 | 22.3 | -0.155727 | 0.0044843 |
| *Samia cynthia ricini* | 11196 | 32.6 | 10.7 | 11.1 | 45.6 | 78.2 | 21.8 | -0.16624 | 0.0183486 |
| *Antheraea yamamai* | 11187 | 32.9 | 10.4 | 10.7 | 46 | 78.9 | 21.1 | -0.166033 | 0.014218 |
| *Eriogyna pyretorum* | 11193 | 33.1 | 10.1 | 10.5 | 46.3 | 79.4 | 20.6 | -0.166247 | 0.0194175 |
| *Saturnia boisduvalii* | 11202 | 33 | 10.1 | 10.8 | 46.1 | 79.1 | 20.9 | -0.165613 | 0.0334928 |
| *Manduca sexta* | 11154 | 34.3 | 9.4 | 10.4 | 45.9 | 80.2 | 19.8 | -0.144638 | 0.0505051 |
| *Antheraea pernyi* | 11196 | 32.7 | 10.6 | 10.9 | 45.8 | 78.5 | 21.5 | -0.166879 | 0.0139535 |
| *Sphinx morio* | 11148 | 34 | 9.7 | 10.5 | 45.8 | 79.8 | 20.2 | -0.14787 | 0.039604 |
| *D. punctatus04* | 11181 | 33.6 | 11 | 11.4 | 44 | 77.6 | 22.4 | -0.134021 | 0.0178571 |
| *D. punctatus05* | 11181 | 33.6 | 11.2 | 11.3 | 43.9 | 77.5 | 22.5 | -0.132903 | 0.0044444 |
| *D. spectabilis02* | 11178 | 33.4 | 11.1 | 11.5 | 44 | 77.4 | 22.6 | -0.136951 | 0.0176991 |
| *D. spectabilis13* | 11178 | 33.4 | 11.1 | 11.5 | 44 | 77.4 | 22.6 | -0.136951 | 0.0176991 |
| *D. tabulaeformis06* | 11178 | 33.6 | 11 | 11.4 | 44 | 77.6 | 22.4 | -0.134021 | 0.0178571 |
| *D. tabulaeformis38* | 11178 | 33.5 | 11.1 | 11.4 | 43.9 | 77.4 | 22.5 | -0.134367 | 0.0133333 |
| *D. punctatus_ws03* | 11181 | 33.5 | 11.1 | 11.5 | 43.9 | 77.4 | 22.6 | -0.134367 | 0.0176991 |
| *D. punctatus_ws06* | 11181 | 33.5 | 11 | 11.5 | 44 | 77.5 | 22.5 | -0.135484 | 0.0222222 |
| tRNA gene |  |  |  |  |  |  |  |  |  |
| *Bombyx mandarina* | 1480 | 41.9 | 10.6 | 7.8 | 39.7 | 81.6 | 18.4 | 0.026961 | -0.152174 |
| *Bombyx mori* | 1468 | 42.1 | 10.7 | 7.9 | 39.3 | 81.4 | 18.6 | 0.034398 | -0.150538 |
| *Actias selene* | 1459 | 40.4 | 11.2 | 8.2 | 40.2 | 80.6 | 19.4 | 0.002481 | -0.154639 |
| *Rondotia menciana* | 1485 | 41.1 | 10.9 | 8.1 | 39.9 | 81 | 19 | 0.014815 | -0.147368 |
| *Attacus atlas* | 1436 | 41.2 | 10.7 | 8.1 | 40 | 81.2 | 18.8 | 0.014778 | -0.138298 |
| *Samia cynthia ricini* | 1463 | 40.6 | 11.2 | 8.2 | 40 | 80.6 | 19.4 | 0.007444 | -0.154639 |
| *Antheraea yamamai* | 1473 | 41.1 | 10.6 | 8.1 | 40.3 | 81.4 | 18.7 | 0.009828 | -0.13369 |
| *Eriogyna pyretorum* | 1477 | 40.4 | 10.3 | 7.9 | 41.5 | 81.9 | 18.2 | -0.013431 | -0.131868 |
| *Saturnia boisduvalii* | 1478 | 40.3 | 10.3 | 7.8 | 41.5 | 81.8 | 18.1 | -0.01467 | -0.138122 |
| *Manduca sexta* | 1484 | 41 | 10.1 | 8.2 | 40.7 | 81.7 | 18.3 | 0.003672 | -0.103825 |
| *Antheraea pernyi* | 1470 | 40.8 | 10.6 | 8 | 40.5 | 81.3 | 18.6 | 0.00369 | -0.139785 |
| *Sphinx morio* | 1463 | 40.6 | 10.2 | 8.2 | 41 | 81.6 | 18.4 | -0.004902 | -0.108696 |
| *D. punctatus04* | 1469 | 41.3 | 11 | 8.2 | 39.6 | 80.9 | 19.2 | 0.021014 | -0.145833 |
| *D. punctatus05* | 1469 | 41.4 | 11 | 8.2 | 39.4 | 80.8 | 19.2 | 0.024752 | -0.145833 |
| *D. spectabilis02* | 1468 | 41.6 | 10.7 | 8.1 | 39.6 | 81.2 | 18.8 | 0.024631 | -0.138298 |
| *D. spectabilis13* | 1468 | 41.6 | 10.7 | 8.1 | 39.6 | 81.2 | 18.8 | 0.024631 | -0.138298 |
| *D. tabulaeformis06* | 1468 | 41.5 | 11 | 8 | 39.5 | 81 | 19 | 0.024691 | -0.157895 |
| *D. tabulaeformis38* | 1468 | 41.5 | 10.9 | 8 | 39.6 | 81.1 | 18.9 | 0.023428 | -0.153439 |
| *D. punctatus_ws03* | 1469 | 41.4 | 11 | 8.1 | 39.5 | 80.9 | 19.1 | 0.023486 | -0.151832 |
| *D. punctatus_ws06* | 1468 | 41.5 | 11 | 8 | 39.5 | 81 | 19 | 0.024691 | -0.157895 |
| rrnS |  |  |  |  |  |  |  |  |  |
| *Bombyx mandarina* | 783 | 43 | 9.6 | 4.5 | 42.9 | 85.9 | 14.1 | 0.001164 | -0.361702 |
| *Bombyx mori* | 783 | 42.8 | 10 | 4.5 | 42.8 | 85.6 | 14.5 | 0 | -0.37931 |
| *Actias selene* | 762 | 39.4 | 10.9 | 5.1 | 44.6 | 84 | 16 | -0.061905 | -0.3625 |
| *Rondotia menciana* | 782 | 42.2 | 10.9 | 4.7 | 42.2 | 84.4 | 15.6 | 0 | -0.397436 |
| *Attacus atlas* | 777 | 40.4 | 10.6 | 6.3 | 42.7 | 83.1 | 16.9 | -0.027677 | -0.254438 |
| *Samia cynthia ricini* | 779 | 40.1 | 11.3 | 4.9 | 43.8 | 83.9 | 16.2 | -0.0441 | -0.395062 |
| *Antheraea yamamai* | 776 | 40.1 | 10.4 | 5.2 | 44.3 | 84.4 | 15.6 | -0.049763 | -0.333333 |
| *Eriogyna pyretorum* | 778 | 38.7 | 10.4 | 5.1 | 45.8 | 84.5 | 15.5 | -0.084024 | -0.341935 |
| *Saturnia boisduvalii* | 774 | 39.7 | 10.9 | 5 | 44.4 | 84.1 | 15.9 | -0.055886 | -0.371069 |
| *Manduca sexta* | 777 | 41.3 | 9.7 | 4.6 | 44.4 | 85.7 | 14.3 | -0.036173 | -0.356643 |
| *Antheraea pernyi* | 775 | 40 | 10.8 | 5 | 44.1 | 84.1 | 15.8 | -0.048751 | -0.367089 |
| *Sphinx morio* | 773 | 42 | 10.1 | 4.7 | 43.2 | 85.2 | 14.8 | -0.014085 | -0.364865 |
| *D. punctatus04* | 779 | 42 | 10.4 | 4.6 | 43 | 85 | 15 | -0.011765 | -0.386667 |
| *D. punctatus05* | 780 | 41.9 | 10.6 | 4.6 | 42.8 | 84.7 | 15.2 | -0.010626 | -0.394737 |
| *D. spectabilis02* | 781 | 40.7 | 10.1 | 4.7 | 44.4 | 85.1 | 14.8 | -0.043478 | -0.364865 |
| *D. spectabilis13* | 779 | 40.8 | 10.1 | 4.7 | 44.3 | 85.1 | 14.8 | -0.041128 | -0.364865 |
| *D. tabulaeformis06* | 778 | 41.9 | 10.7 | 4.6 | 42.8 | 84.7 | 15.3 | -0.010626 | -0.398693 |
| *D. tabulaeformis38* | 779 | 41.2 | 10.4 | 4.6 | 43.8 | 85 | 15 | -0.030588 | -0.386667 |
| *D. punctatus_ws03* | 779 | 41.8 | 10.8 | 4.6 | 42.7 | 84.5 | 15.4 | -0.010651 | -0.402597 |
| *D. punctatus_ws06* | 779 | 41.8 | 10.4 | 4.6 | 43.1 | 84.9 | 15 | -0.015312 | -0.386667 |
| rrnL |  |  |  |  |  |  |  |  |  |
| *Bombyx mandarina* | 1377 | 44.4 | 10.5 | 4.7 | 40.4 | 84.8 | 15.2 | 0.04717 | -0.381579 |
| *Bombyx mori* | 1375 | 44.3 | 11 | 4.7 | 40.1 | 84.4 | 15.7 | 0.049763 | -0.401274 |
| *Actias selene* | 1364 | 40.2 | 11.5 | 4.9 | 43.3 | 83.5 | 16.4 | -0.037126 | -0.402439 |
| *Rondotia menciana* | 1365 | 43.5 | 11.7 | 4.9 | 39.9 | 83.4 | 16.6 | 0.043165 | -0.409639 |
| *Attacus atlas* | 1368 | 42.5 | 10.4 | 4.8 | 42.3 | 84.8 | 15.2 | 0.002358 | -0.368421 |
| *Samia cynthia ricini* | 1358 | 41.7 | 11 | 4.9 | 42.3 | 84 | 15.9 | -0.007143 | -0.383648 |
| *Antheraea yamamai* | 1380 | 41 | 11 | 5 | 43 | 84 | 16 | -0.02381 | -0.375 |
| *Eriogyna pyretorum* | 1338 | 42.6 | 10.8 | 4.6 | 42 | 84.6 | 15.4 | 0.007092 | -0.402597 |
| *Saturnia boisduvalii* | 1391 | 41.1 | 10.2 | 5 | 43.6 | 84.7 | 15.2 | -0.029516 | -0.342105 |
| *Manduca sexta* | 1391 | 41.4 | 9.8 | 5 | 43.9 | 85.3 | 14.8 | -0.029308 | -0.324324 |
| *Antheraea pernyi* | 1369 | 41.3 | 11.3 | 4.8 | 42.5 | 83.8 | 16.1 | -0.01432 | -0.403727 |
| *Sphinx morio* | 1379 | 41.6 | 10.5 | 4.9 | 43 | 84.6 | 15.4 | -0.016548 | -0.363636 |
| *D. punctatus04* | 1461 | 44.6 | 11.1 | 4.3 | 40 | 84.6 | 15.4 | 0.054374 | -0.441558 |
| *D. punctatus05* | 1452 | 44.4 | 11.2 | 4.3 | 40 | 84.4 | 15.5 | 0.052133 | -0.445161 |
| *D. spectabilis02* | 1454 | 44.1 | 11.6 | 4.5 | 39.8 | 83.9 | 16.1 | 0.051251 | -0.440994 |
| *D. spectabilis13* | 1454 | 44.1 | 11.6 | 4.5 | 39.8 | 83.9 | 16.1 | 0.051251 | -0.440994 |
| *D. tabulaeformis06* | 1459 | 44.8 | 11 | 4.2 | 40 | 84.8 | 15.2 | 0.056604 | -0.447368 |
| *D. tabulaeformis38* | 1456 | 44.8 | 11.1 | 4.3 | 39.8 | 84.6 | 15.4 | 0.059102 | -0.441558 |
| *D. punctatus_ws03* | 1462 | 44.9 | 11 | 4.2 | 39.9 | 84.8 | 15.2 | 0.058962 | -0.447368 |
| *D. punctatus_ws06* | 1462 | 44.9 | 10.9 | 4.2 | 39.9 | 84.8 | 15.1 | 0.058962 | -0.443709 |
| AT region |  |  |  |  |  |  |  |  |  |
| *Bombyx mandarina* | 747 | 45.5 | 2.4 | 2.4 | 49.7 | 95.2 | 4.8 | -0.044118 | 0 |
| *Bombyx mori* | 499 | 44.7 | 3 | 1.6 | 50.7 | 95.4 | 4.6 | -0.062893 | -0.304348 |
| *Actias selene* | 339 | 43.1 | 6.2 | 5.9 | 44.8 | 87.9 | 12.1 | -0.01934 | -0.024793 |
| *Rondotia menciana* | 357 | 43.7 | 5.6 | 3.4 | 47.3 | 91 | 9 | -0.03956 | -0.244444 |
| *Attacus atlas* | 359 | 41.5 | 5.3 | 4.5 | 48.7 | 90.2 | 9.8 | -0.079823 | -0.081633 |
| *Samia cynthia ricini* | 361 | 44.3 | 5.5 | 3.6 | 46.5 | 90.8 | 9.1 | -0.024229 | -0.208791 |
| *Antheraea yamamai* | 334 | 41.6 | 6.9 | 3.6 | 47.9 | 89.5 | 10.5 | -0.070391 | -0.314286 |
| *Eriogyna pyretorum* | 358 | 42.2 | 5.3 | 2.5 | 50 | 92.2 | 7.8 | -0.084599 | -0.358974 |
| *Saturnia boisduvalii* | 330 | 42.1 | 6.4 | 2.1 | 49.4 | 91.5 | 8.5 | -0.079781 | -0.505882 |
| *Manduca sexta* | 324 | 45.1 | 3.1 | 1.5 | 50.3 | 95.4 | 4.6 | -0.054507 | -0.347826 |
| *Antheraea pernyi* | 552 | 41.1 | 5.4 | 4.2 | 49.3 | 90.4 | 9.6 | -0.090708 | -0.125 |
| *Sphinx morio* | 316 | 44.3 | 4.7 | 2.5 | 48.4 | 92.7 | 7.2 | -0.044229 | -0.305556 |
| *D. punctatus04* | 320 | 43.1 | 4.4 | 2.8 | 49.7 | 92.8 | 7.2 | -0.071121 | -0.222222 |
| *D. punctatus05* | 320 | 42.2 | 4.7 | 3.4 | 49.7 | 91.9 | 8.1 | -0.08161 | -0.160494 |
| *D. spectabilis02* | 320 | 42.8 | 4.4 | 2.2 | 50.6 | 93.4 | 6.6 | -0.083512 | -0.333333 |
| *D. spectabilis13* | 320 | 42.8 | 4.4 | 2.2 | 50.6 | 93.4 | 6.6 | -0.083512 | -0.333333 |
| *D. tabulaeformis06* | 320 | 43.1 | 4.4 | 2.8 | 49.7 | 92.8 | 7.2 | -0.071121 | -0.222222 |
| *D. tabulaeformis38* | 320 | 43.1 | 4.4 | 2.8 | 49.7 | 92.8 | 7.2 | -0.071121 | -0.222222 |
| *D. punctatus_ws03* | 320 | 43.1 | 4.7 | 2.8 | 49.4 | 92.5 | 7.5 | -0.068108 | -0.253333 |
| *D. punctatus_ws06* | 320 | 43.1 | 4.7 | 2.8 | 49.4 | 92.5 | 7.5 | -0.068108 | -0.253333 |
