# Supplementary material for: Mitochondrial phylogenomics and genetic relationships of closely related pine moth (Lasiocampidae: Dendrolimus) species in China, using whole mitochondrial genomes
Source: BMC Genomics. 2015 Jun 4;16(1):428. doi: 10.1186/s12864-015-1566-5 (PMC4455531; doi:10.1186/s12864-015-1566-5)
Supplement: Additional file 9: — (a). Codon usage of eight Dendrolimus mitochondrial genomes. Numbers above the column refer to the number of codons. The total number of codons of each individual is given. CDspT stands for codons per thousand codons. Codon Families are provided on the x axis. (b). The relative synonymous codon usage (RSCU) of eight Dendrolimus mitochondrial genomes. Codon Families are provided on the x axis. Codons that are absent in the mitochondrial genomes are marked at the top of columns. (c). Codon usage of Bombycoidea mitochondrial genomes that are published in GenBank. Numbers above the column refer to the number of codons. The total number of codons of each individual is given. CDspT stands for codons per thousand codons. Codon Families are provided on the x axis. (d). Codon usage of Bombycoidea mitochondrial genomes that are published in GenBank. Codon Families are provided on the x axis. Codons that are absent in the mitochondrial genomes are marked at the top of columns. [file 12864_2015_1566_MOESM9_ESM.docx]

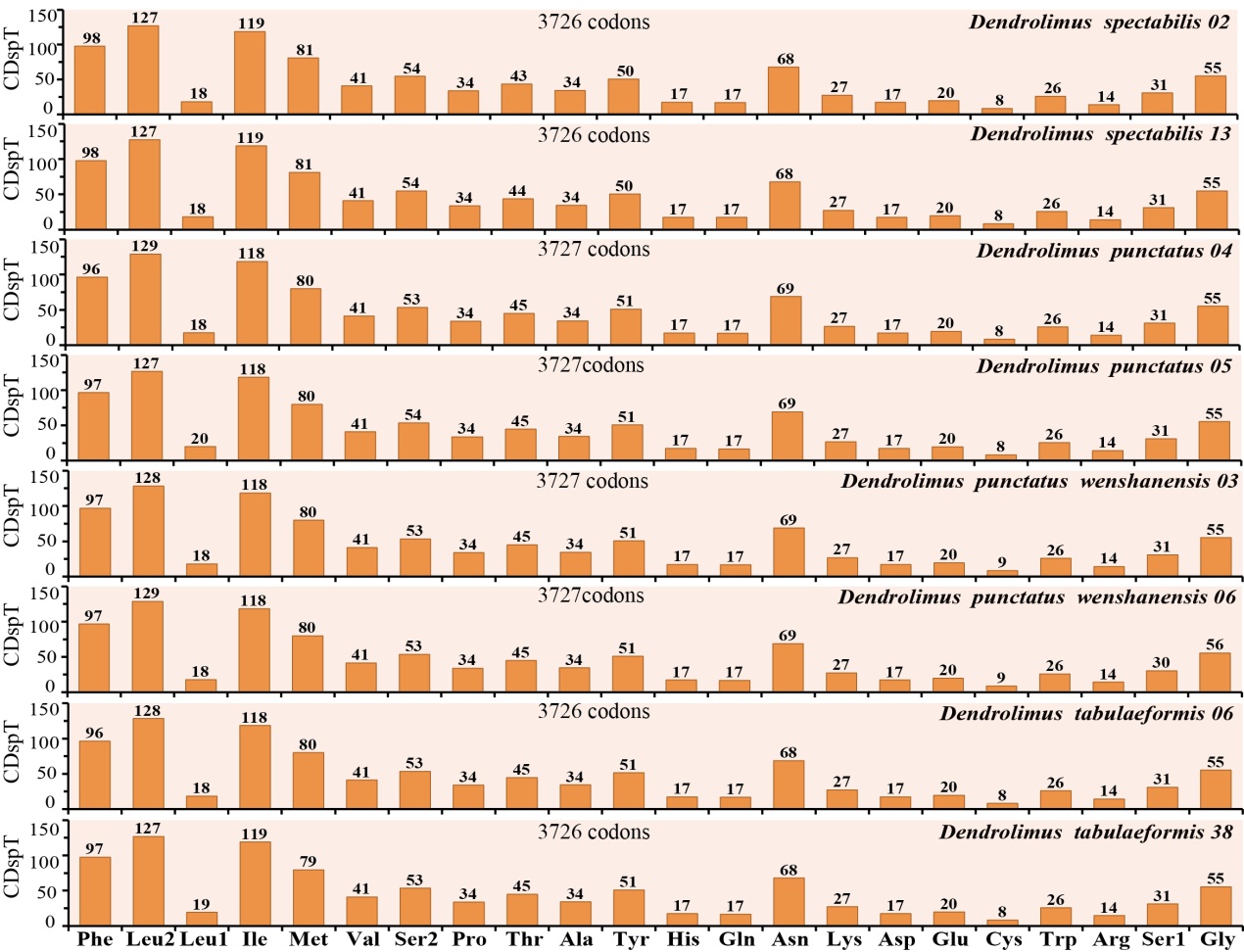


Additional file 9 (a) Codon usage of eight *Dendrolimus* mitochondrial genomes. Numbers above the column refer to the number of codons. The total number of codons of each individual is given. CDspT stands for codons per thousand codons. Codon Families are provided on the x axis.


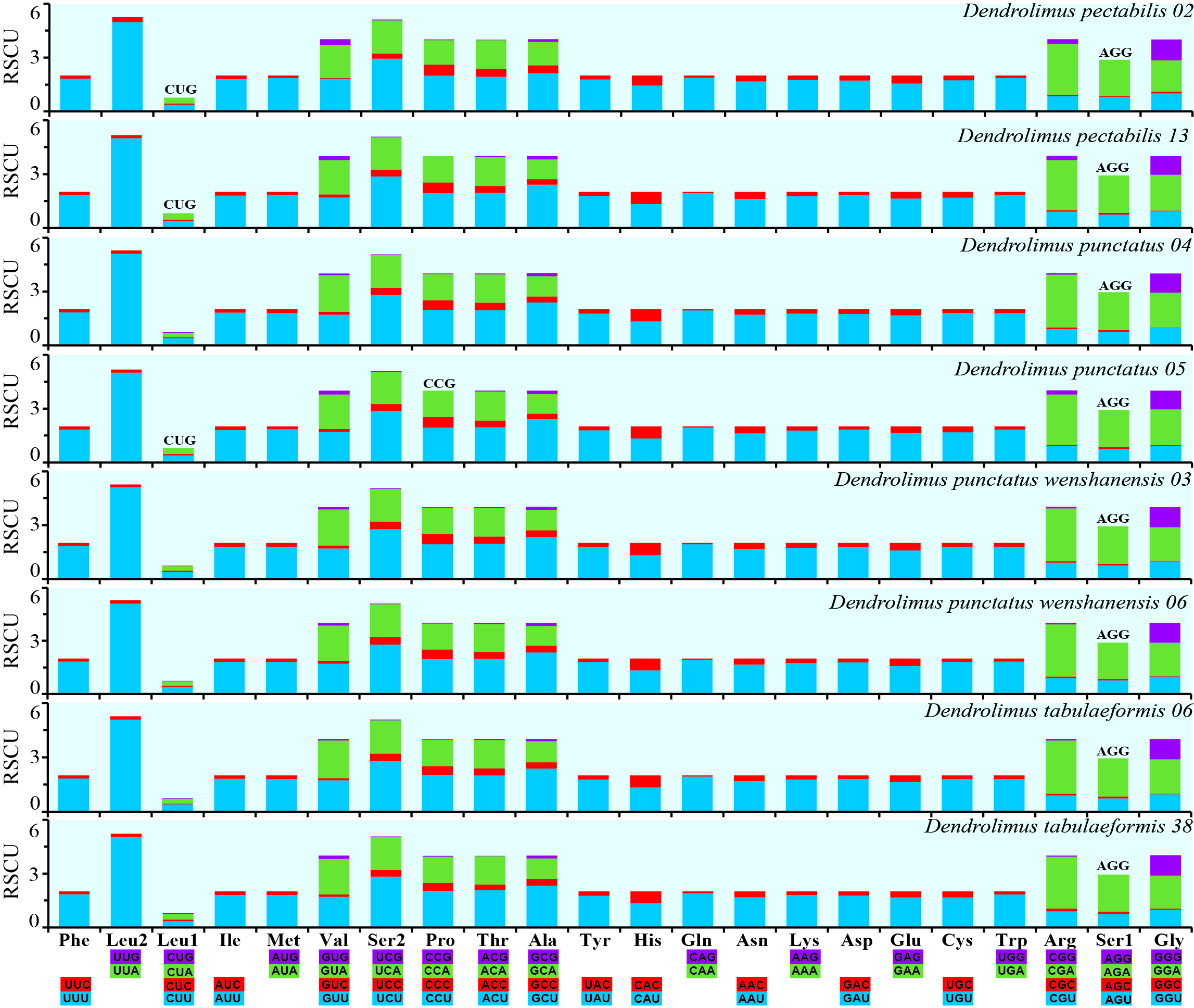


Additional file 9 (b) The relative synonymous codon usage (RSCU) of eight Dendrolimus mitochondrial genomes. Codon Families are provided on the x axis. Codons that are absent in the mitochondrial genomes are marked at the top of columns.


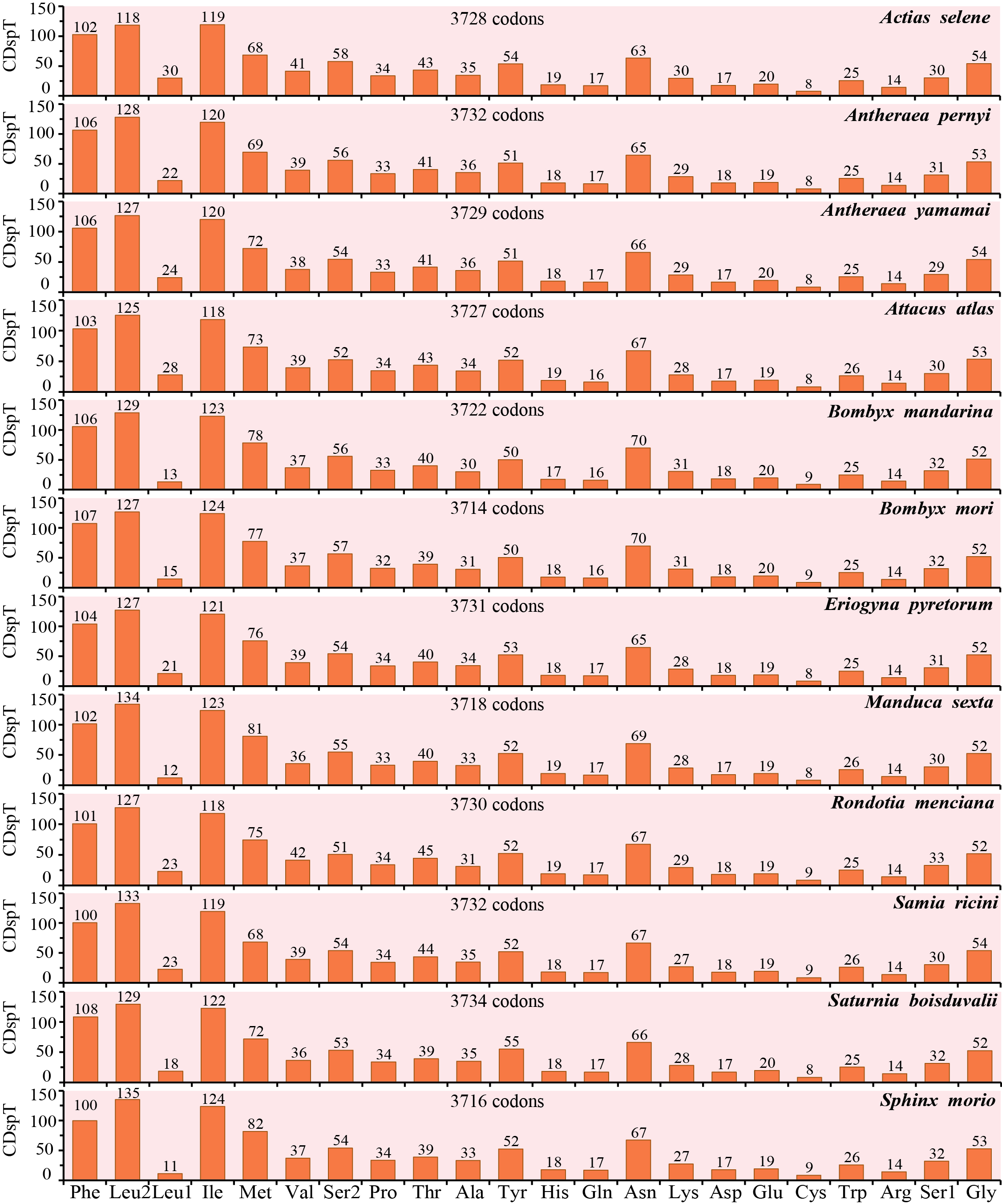


Additional file 9 (c) Codon usage of Bombycoidea mitochondrial genomes that are published in GenBank. Numbers above the column refer to the number of codons. The total number of codons of each individual is given. CDspT stands for codons per thousand codons. Codon Families are provided on the x axis.


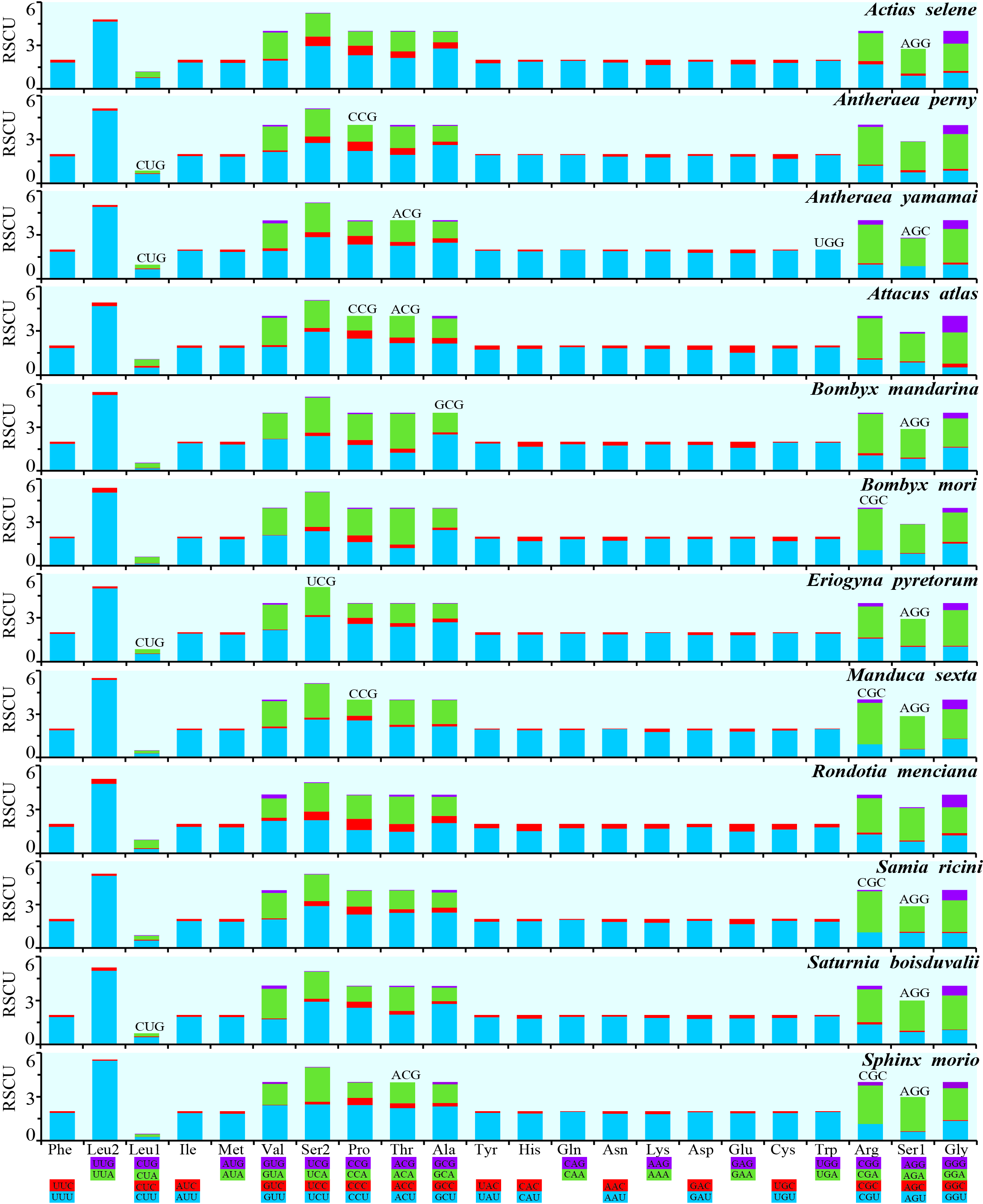


Additional file 9 (d) Codon usage of Bombycoidea mitochondrial genomes that are published in GenBank. Codon Families are provided on the x axis. Codons that are absent in the mitochondrial genomes are marked at the top of columns.
