# Supplementary material for: Mitochondrial phylogenomics and genetic relationships of closely related pine moth (Lasiocampidae: Dendrolimus) species in China, using whole mitochondrial genomes
Source: BMC Genomics. 2015 Jun 4;16(1):428. doi: 10.1186/s12864-015-1566-5 (PMC4455531; doi:10.1186/s12864-015-1566-5)
Supplement: Additional file 10: — Genetic distance used HKY85 model for whole mitochondrial genomes and different components. [file 12864_2015_1566_MOESM10_ESM.docx]

| Additional file 10 Genetic distance used selected best-fitting HKY85 model for whole mitochondrial genomes and different components. | | | |
| --- | --- | --- | --- |
| **All** | Range-1 | Average distance | Range-2 |
| *D. punctatus-D. punctatus* | 0.0157 | 0.0157 | 0.0157 |
| *D. punctatus-D. punctatus_ws* | 0.0046 | 0.0104 | 0.0161 |
| *D. punctatus-D. tabulaeformis* | 0.0040 | 0.0129 | 0.0195 |
| *D. punctatus-D. spectabilis* | 0.0445 | 0.0450 | 0.0454 |
| **37gene** | Range-1 | Average distance | Range-2 |
| *D. punctatus-D. punctatus* | 0.0154 | 0.0154 | 0.0154 |
| *D. punctatus-D. punctatus_ws* | 0.0046 | 0.0103 | 0.0160 |
| *D. punctatus-D. tabulaeformis* | 0.0040 | 0.0129 | 0.0194 |
| *D. punctatus-D. spectabilis* | 0.0448 | 0.0451 | 0.0454 |
| **13PCGs** | Range-1 | Average distance | Range-2 |
| *D. punctatus-D. punctatus* | 0.0183 | 0.0183 | 0.0183 |
| *D. punctatus-D. punctatus_ws* | 0.0054 | 0.0122 | 0.0189 |
| *D. punctatus-D. tabulaeformis* | 0.0047 | 0.0154 | 0.0230 |
| *D. punctatus-D. spectabilis* | 0.0504 | 0.0509 | 0.0515 |
| **ATP6** | Range-1 | Average distance | Range-2 |
| *D. punctatus-D. punctatus* | 0.0255 | 0.0255 | 0.0255 |
| *D. punctatus-D. punctatus_ws* | 0.0030 | 0.0135 | 0.0240 |
| *D. punctatus-D. tabulaeformis* | 0.0059 | 0.0244 | 0.0378 |
| *D. punctatus-D. spectabilis* | 0.0519 | 0.0575 | 0.0630 |
| **COX1** | Range-1 | Average distance | Range-2 |
| *D. punctatus-D. punctatus* | 0.0225 | 0.0225 | 0.0225 |
| *D. punctatus-D. punctatus_ws* | 0.0059 | 0.0139 | 0.0219 |
| *D. punctatus-D. tabulaeformis* | 0.0066 | 0.0172 | 0.0232 |
| *D. punctatus-D. spectabilis* | 0.0507 | 0.0507 | 0.0507 |
| **ND2** | Range-1 | Average distance | Range-2 |
| *D. punctatus-D. punctatus* | 0.0171 | 0.0171 | 0.0171 |
| *D. punctatus-D. punctatus_ws* | 0.0050 | 0.0105 | 0.0160 |
| *D. punctatus-D. tabulaeformis* | 0.0060 | 0.0105 | 0.0150 |
| *D. punctatus-D. spectabilis* | 0.0439 | 0.0466 | 0.0492 |
| **ND4** | Range-1 | Average distance | Range-2 |
| *D. punctatus-D. punctatus* | 0.0143 | 0.0143 | 0.0143 |
| *D. punctatus-D. punctatus_ws* | 0.0030 | 0.0094 | 0.0166 |
| *D. punctatus-D. tabulaeformis* | 0.0022 | 0.0083 | 0.0143 |
| *D. punctatus-D. spectabilis* | 0.0422 | 0.0446 | 0.0470 |
| **ND4L** | Range-1 | Average distance | Range-2 |
| *D. punctatus-D. punctatus* | 0.0068 | 0.0068 | 0.0068 |
| *D. punctatus-D. punctatus_ws* | 0.0068 | 0.0103 | 0.0137 |
| *D. punctatus-D. tabulaeformis* | 0.0000 | 0.0051 | 0.0103 |
| *D. punctatus-D. spectabilis* | 0.0313 | 0.0348 | 0.0384 |
| **ND5** | Range-1 | Average distance | Range-2 |
| *D. punctatus-D. punctatus* | 0.0221 | 0.0221 | 0.0221 |
| *D. punctatus-D. punctatus_ws* | 0.0040 | 0.0131 | 0.0221 |
| *D. punctatus-D. tabulaeformis* | 0.0040 | 0.0134 | 0.0227 |
| *D. punctatus-D. spectabilis* | 0.0449 | 0.0468 | 0.0486 |
| **ND6** | Range-1 | Average distance | Range-2 |
| *D. punctatus-D. punctatus* | 0.0191 | 0.0191 | 0.0191 |
| *D. punctatus-D. punctatus_ws* | 0.0019 | 0.0124 | 0.0229 |
| *D. punctatus-D. tabulaeformis* | 0.0038 | 0.0114 | 0.0191 |
| *D. punctatus-D. spectabilis* | 0.0649 | 0.0669 | 0.0690 |
| **tRNA** | Range-1 | Average distance | Range-2 |
| *D. punctatus-D. punctatus* | 0.0034 | 0.0034 | 0.0034 |
| *D. punctatus-D. punctatus_ws* | 0.0014 | 0.0024 | 0.0034 |
| *D. punctatus-D. tabulaeformis* | 0.0014 | 0.0020 | 0.0027 |
| *D. punctatus-D. spectabilis* | 0.0138 | 0.0141 | 0.0145 |
| **rRNA** | Range-1 | Average distance | Range-2 |
| *D. punctatus-D. punctatus* | 0.0086 | 0.0086 | 0.0086 |
| *D. punctatus-D. punctatus_ws* | 0.0027 | 0.0061 | 0.0096 |
| *D. punctatus-D. tabulaeformis* | 0.0027 | 0.0075 | 0.0123 |
| *D. punctatus-D. spectabilis* | 0.0356 | 0.0366 | 0.0376 |
| **Noncoding** | Range-1 | Average distance | Range-2 |
| *D. punctatus-D. punctatus* | 0.0418 | 0.0418 | 0.0418 |
| *D. punctatus-D. punctatus_ws* | 0.0042 | 0.0208 | 0.0373 |
| *D. punctatus-D. tabulaeformis* | 0.0021 | 0.0208 | 0.0396 |
| *D. punctatus-D. spectabilis* | 0.0888 | 0.0907 | 0.0926 |
| **ATP8** | Range-1 | Average distance | Range-2 |
| *D. punctatus-D. punctatus* | 0.0063 | 0.0063 | 0.0063 |
| *D. punctatus-D. punctatus_ws* | 0.0000 | 0.0032 | 0.0063 |
| *D. punctatus-D. tabulaeformis* | 0.0000 | 0.0128 | 0.0256 |
| *D. punctatus-D. spectabilis* | 0.0454 | 0.0487 | 0.0521 |
| **COX2** | Range-1 | Average distance | Range-2 |
| *D. punctatus-D. punctatus* | 0.0223 | 0.0223 | 0.0223 |
| *D. punctatus-D. punctatus_ws* | 0.0088 | 0.0171 | 0.0269 |
| *D. punctatus-D. tabulaeformis* | 0.0059 | 0.0247 | 0.0391 |
| *D. punctatus-D. spectabilis* | 0.0485 | 0.0524 | 0.0563 |
| **COX3** | Range-1 | Average distance | Range-2 |
| *D. punctatus-D. punctatus* | 0.0167 | 0.0167 | 0.0167 |
| *D. punctatus-D. punctatus_ws* | 0.0076 | 0.0141 | 0.0219 |
| *D. punctatus-D. tabulaeformis* | 0.0051 | 0.0187 | 0.0284 |
| *D. punctatus-D. spectabilis* | 0.0484 | 0.0491 | 0.0498 |
| **Cytb** | Range-1 | Average distance | Range-2 |
| *D. punctatus-D. punctatus* | 0.0167 | 0.0167 | 0.0167 |
| *D. punctatus-D. punctatus_ws* | 0.0079 | 0.0123 | 0.0158 |
| *D. punctatus-D. tabulaeformis* | 0.0052 | 0.0199 | 0.0311 |
| *D. punctatus-D. spectabilis* | 0.0731 | 0.075 | 0.0769 |
| **ND1** | Range-1 | Average distance | Range-2 |
| *D. punctatus-D. punctatus* | 0.0170 | 0.0170 | 0.017 |
| *D. punctatus-D. punctatus_ws* | 0.0042 | 0.0122 | 0.0191 |
| *D. punctatus-D. tabulaeformis* | 0.0053 | 0.0197 | 0.0278 |
| *D. punctatus-D. spectabilis* | 0.0387 | 0.0387 | 0.0387 |
| **ND3** | Range-1 | Average distance | Range-2 |
| *D. punctatus-D. punctatus* | 0.0057 | 0.0057 | 0.0057 |
| *D. punctatus-D. punctatus_ws* | 0.0028 | 0.0043 | 0.0057 |
| *D. punctatus-D. tabulaeformis* | 0.0028 | 0.0064 | 0.0085 |
| *D. punctatus-D. spectabilis* | 0.0437 | 0.0452 | 0.0468 |
