# Supplementary material for: Mitochondrial phylogenomics and genetic relationships of closely related pine moth (Lasiocampidae: Dendrolimus) species in China, using whole mitochondrial genomes
Source: BMC Genomics. 2015 Jun 4;16(1):428. doi: 10.1186/s12864-015-1566-5 (PMC4455531; doi:10.1186/s12864-015-1566-5)
Supplement: Additional file 11: — Genetic distance used selected best-fitting GTR model measures for the COI barcoding region of the additional dataset. [file 12864_2015_1566_MOESM11_ESM.docx]

| Additional file 11 Genetic distance used selected best-fitting GTR model measures for the COI barcoding region of the additional dataset. | | | |
| --- | --- | --- | --- |
| Species | CI-1 | Average distance | CI-2 |
| *D. punctatus*-*D. punctatus* | 0.01245 | 0.01251 | 0.01257 |
| *D. punctatus-D. punctatus_ws* | 0.01283 | 0.01322 | 0.01361 |
| *D. punctatus-D. tabulaeformis* | 0.01306 | 0.01319 | 0.01331 |
| *D. punctatus-D. spectabilis* | 0.04833 | 0.04868 | 0.04903 |
