# Supplementary material for: Mitochondrial phylogenomics and genetic relationships of closely related pine moth (Lasiocampidae: Dendrolimus) species in China, using whole mitochondrial genomes
Source: BMC Genomics. 2015 Jun 4;16(1):428. doi: 10.1186/s12864-015-1566-5 (PMC4455531; doi:10.1186/s12864-015-1566-5)
Supplement: Additional file 12: — Intergenic spacer locations and nucleotides of eight sequenced pine moths. [file 12864_2015_1566_MOESM12_ESM.docx]

Additional file 12 Intergenic spacer locations and nucleotides of eight sequenced pine moths.

| Location | *D. spectabilis02* | *D. spectabilis13* | *D. tabulaeformis06* | *D. tabulaeformis38* | *D. punctatus04* | *D. punctatus05* | *D. punctatus_ws03* | *D. punctatus_ws06* |
| --- | --- | --- | --- | --- | --- | --- | --- | --- |
| trnM-trnI | 3 | 3 | 3 | 3 | 3 | 3 | 3 | 3 |
| trnQ-nd2 | 58 | 58 | 58 | 58 | 58 | 58 | 58 | 58 |
| trnY-cox1 | 25 | 25 | 34 | 34 | 27 | 34 | 34 | 34 |
| trnK-trnD | 3 | 3 | 3 | 3 | 3 | 3 | 3 | 3 |
| atp6-cox3 | 11 | 11 | 15 | 15 | 15 | 14 | 15 | 15 |
| cox3-trnG | 2 | 2 | 2 | 2 | 2 | 2 | 2 | 2 |
| trnA-trnR | 20 | 20 | 15 | 15 | 15 | 15 | 15 | 15 |
| trnR-trnN | 4 | 4 | 4 | 4 | 4 | 4 | 4 | 4 |
| trnN-trnS(AGN) | 18 | 18 | 11 | 11 | 11 | 13 | 11 | 11 |
| trnE-trnF | 8 | 8 | 4 | 4 | 4 | 4 | 4 | 4 |
| trnF-nd5 | 3 | 3 | 2 | 2 | 2 | 3 | 2 | 2 |
| nd4-nd4l | 23 | 23 | 24 | 24 | 24 | 19 | 24 | 24 |
| nd4l-trnT | 7 | 7 | 7 | 7 | 7 | 7 | 7 | 7 |
| trnP-nd6 | 8 | 8 | 8 | 8 | 8 | 8 | 8 | 8 |
| nd6-cytb | 4 | 4 | 4 | 4 | 4 | 4 | 4 | 4 |
| cytb-trnS(UCN) | 3 | 3 | 3 | 3 | 3 | 3 | 3 | 3 |
| nd1-trnL(CUN) | 1 | 1 | 1 | 1 | 1 | 1 | 1 | 1 |
| Total | 201 | 201 | 198 | 198 | 191 | 195 | 198 | 198 |
