# Supplementary material for: Mitochondrial phylogenomics and genetic relationships of closely related pine moth (Lasiocampidae: Dendrolimus) species in China, using whole mitochondrial genomes
Source: BMC Genomics. 2015 Jun 4;16(1):428. doi: 10.1186/s12864-015-1566-5 (PMC4455531; doi:10.1186/s12864-015-1566-5)
Supplement: Additional file 13: — Six intergenic spacers in the eight sequenced mitochondrial genomes. The locations of the intergenic spacers are indicated on the top of the figure. The left side of figure is the abbreviated species name. Spectabilis02 stands for Dendrolimus spectabilis02, Spectabilis13 stands for Dendrolimus spectabilis13, Punctatus04 stands for Dendrolimus punctatus04, Punctatus05 stands for Dendrolimus punctatus05, Punctatus_ws03 stands for Dendrolimus punctatus wenshanensis03, Punctatus_ws06 stands for Dendrolimus punctatus wenshanensis06, Tabulaeformis06 stands for Dendrolimus tabulaeformis06, Tabulaeformis38 stands for Dendrolimus tabulaeformis38. Different colors represent different deoxynucleotides. Green represents adenine, purple guanine, blue cytosine and orange thymine. Dashes (-) indicate no nucleotide. [file 12864_2015_1566_MOESM13_ESM.docx]

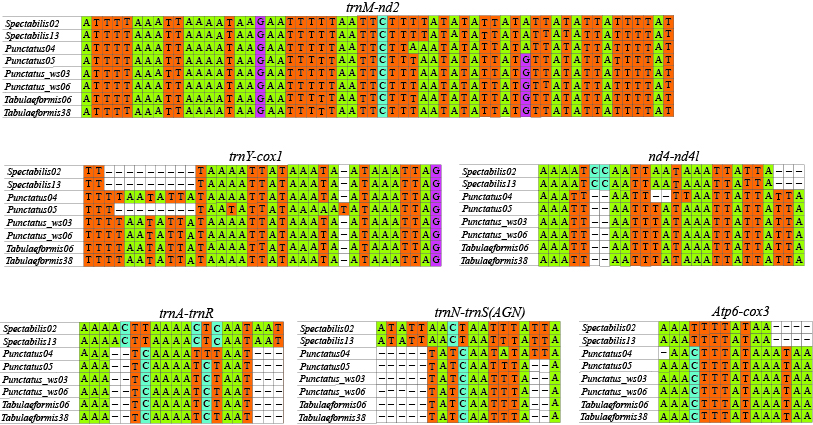


Additional file 13 Six intergenic spacers in the eight sequenced mitochondrial genomes. The locations of the intergenic spacers are indicated on the top of the figure. The left side of figure is the abbreviated species name. *Spectabilis*02 stands for *Dendrolimus spectabilis*02*, Spectabilis*13 stands for *Dendrolimus spectabilis*13*, Punctatus*04 stands for *Dendrolimus punctatus*04*, Punctatus*05 *stands for Dendrolimus punctatus*05*, Punctatus_ws*03 *stands for Dendrolimus punctatus wenshanensis*03*, Punctatus_ws*06 *stands for Dendrolimus punctatus wenshanensis*06*, Tabulaeformis*06 *stands for Dendrolimus tabulaeformis*06*, Tabulaeformis*38 *stands for Dendrolimus tabulaeformis*38*.* Different colors represent different deoxynucleotides. Green represents adenine, purple guanine, blue cytosine and orange thymine. Dashes (-) indicate no nucleotide.
